# Evaluation of hydrophobically associating cationic starch-based flocculants in sludge dewatering

**DOI:** 10.1038/s41598-021-91323-y

**Published:** 2021-06-03

**Authors:** Pan Hu, Shaohang Shen, Hu Yang

**Affiliations:** State Key Laboratory of Pollution Control and Resource Reuse, School of the Environment, Nanjing University, Nanjing, 210023 People’s Republic of China

**Keywords:** Pollution remediation, Polymers, Sustainability

## Abstract

Two series of binary graft cationic starch-based flocculants (CS-DMCs and CS-DMLs) with different hydrophilicity and charge density (CD) were prepared by graft copolymerization of acrylamide with 2-(Methacryloyloxy)-*N*,*N*,*N*-trimethylethanaminium chloride and methacrylic acid 2-(benzyldimethylaminio) ethyl chloride, respectively, on the starch (St) backbone. The sludge dewatering performance of CS-DMCs and CS-DMLs were evaluated and compared based on the changes in filter cake moisture content (FCMC), specific resistance of filtration (SRF), fractions and components of extracellular polymeric substances, and various physiochemical characteristics of sludge flocs and cakes. Increase in CD of the St-based flocculants caused improved sludge dewaterability. Under the similar CD, CS-DML with relatively high hydrophobicity exhibited lower FCMC and SRF, larger and denser sludge flocs, and better permeability of sludge cakes than CS-DMCs due to the synergistic effects of charge neutralization, bridging flocculation and hydrophobic association. Furthermore, a second-order polynomial model on the basis of phenomenological theory was successfully applied to quantitatively evaluate the influences of the two important structural factors of these St-based flocculants, i.e., hydrophobicity and CD, on the sludge dewaterability. The structure–activity relationship of the St-based flocculants in sludge dewatering was obtained according to the theoretic simulation. The dewatering mechanisms was discussed in depth on the basis of the experimental and simulated results; besides, the FCMC and optimal dose can be predicted by the established structure–activity relationship. This current work offered a novel and valuable way to exploit and design of low-cost and high-performance graft natural polymeric flocculants applied in efficient conditioning of sludge.

## Introduction

The management and disposal of sludge has become one of key problems in water and wastewater treatment with accelerated urbanization and stringent environmental regulations^1–3^. Sludge usually contains more than 95% water, in addition to the solid ingredients mainly composed of inorganic colloidal particles, microorganisms, organic debris, and extracellular polymeric substances (EPS)^4,5^. Efficient separation of the solid sludge and water is thus one of the crucial steps to reduce the cost of sludge treatment, transportation, and final disposal^6–8^. Sludge dewatering is a process of water repelling and drainage, and the microstructural characteristics of resultant sludge flocs and mud cakes strongly affect the sludge dewaterability^7,9^. Generally, large and dense resultant sludge flocs and mud cakes with certain mechanical strength and good permeability was usually beneficial to further sludge dewatering^3,7,9^. However, EPS, one of main components of sludge, has the characteristics of strong hydrophilicity due to high negative charges, and is readily to form a stable gelatinous suspension system binding with a huge amount of water^4,5^. It is thus quite difficult to achieve a desired liquid and solid separation effect in sludge^10,11^. Suitable increase in hydrophobicity of sludge might destroy the hydration, form a rigid porous microstructure in sludge cakes and well build water drainage channels originated in the formed hydrophobic regions, and thus improve the sludge dewaterability^12–14^.

Coagulation/flocculation can effectively agglomerate sludge colloids to form large flocs through charge neutralization and bridging flocculation effects, is beneficial to sludge sedimentation, filtration and permeability, and is thus one of the most commonly used sludge conditionings in practical operation^15–17^. The employed coagulants/flocculants are of great significance in coagulation/flocculation^18,19^. Given the critical roles of the microstructural characteristics of resultant sludge cakes in the sludge dewaterability as mentioned above, the ideal sludge conditioners should contain not only hydrophilic groups for improved water solubility and dispersion but also hydrophobic ones to form hydrophobic regions for well building drainage channels. It is thus an interesting topic to design and fabricate hydrophobic modified coagulants/flocculants to further enhance the sludge dewaterability by improvement of the hydrophobicity of sludge surface in the conditioning process, which can be beneficial to destruction of the hydration, formation of the internal rigid porous microstructure, and well building of the water drainage channels in resultant sludge cakes^14,20^.

In addition to traditional inorganic coagulants and synthetic flocculants including polyaluminium chloride, ferric chloride, and polyacrylamide derivatives^11,21–23^, natural polymeric flocculants have recently gained much more attentions due to their wide source, low toxicity, and high efficiency^20,24,25^. Besides, various functional groups including different hydrophilic and hydrophobic ones could be conveniently introduced onto the polymeric backbone to improve the flocculation effect by various chemical modifications including graft copolymerization, esterification and etherification, due to their abundant active functional groups contained^26,27^. Previously introduced functional groups onto the natural polymers were mainly focused on the hydrophilic cationic ones to improve the water solubility, and charge neutralization effect^20,28,29^. However, there was few report on the natural polymeric flocculants as effective sludge conditioners undergone by both hydrophobic and hydrophilic modifications. Besides, the work relevant to the structure–activity relationship between the fine hydrophilic and hydrophobic structures of natural polymeric flocculants and the sludge dewatering performance was seldom investigated, and a few previous studies involved the structural effects of natural polymeric flocculants on dewatering behaviors were mainly limited in qualitative analysis whereas quantitative analysis is little^23,30^.

Graft copolymerization is a simple and popular modification method^28,29^, and starch (St) is one of the most abundant and low-cost natural polymers in nature^26,31,32^. Given the important role of the hydrophobicity of conditioners in improvement of sludge dewaterability, the St-based flocculants (CS-DMCs and CS-DMLs) with different hydrophilicity and charge density (CD) were prepared by graft copolymerization of acrylamide (AM) with 2-(Methacryloyloxy)-*N*,*N*,*N*-trimethylethanaminium chloride (DMC) and methacrylic acid 2-(benzyldimethylaminio) ethyl chloride (DML), respectively, on the St backbone in this work. Fourier transform infrared spectroscopy (FTIR) and ^1^H nuclear magnetic resonance (^1^H NMR) were applied to characterize the molecular structures of those St-based flocculants. The effects of hydrophilicity and CD on the sludge dewaterability of CS-DMCs and CS-DMLs were evaluated and compared on the basis of the changes in filter cake moisture content (FCMC), specific resistance of filtration (SRF), bound water content, zeta potentials, microstructures of sludge cake, properties of sludge flocs, fractions and components of EPS, and compression coefficient. Furthermore, on the basis of phenomenological theory^33^, a mathematical model, i.e., the second-order polynomial model, was applied to simulate the experimental results of sludge dewatering by the starch-based flocculants^26^, and the influences of the two structural factors of the starch-based flocculants on the sludge dewaterability were quantitatively analyzed. Accordingly, the dewatering mechanism was discussed from the molecular levels; besides, the FCMC and optimal dose were tried to predict based on the established model.

## Results and discussion

### Characterization of St-based flocculants

**Scheme 1:**
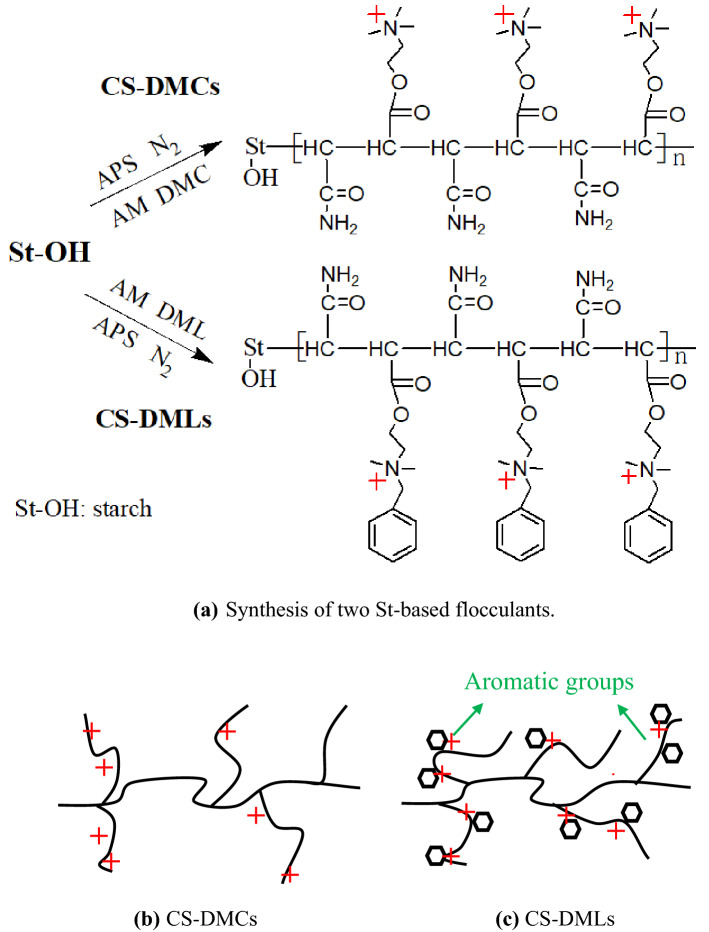
(**a**) Synthesis of two St-based flocculants with different structural morphologies, (**b**) CS-DMCs; and (**c**) CS-DMLs with rich aromatic groups.

**Figure 1:**
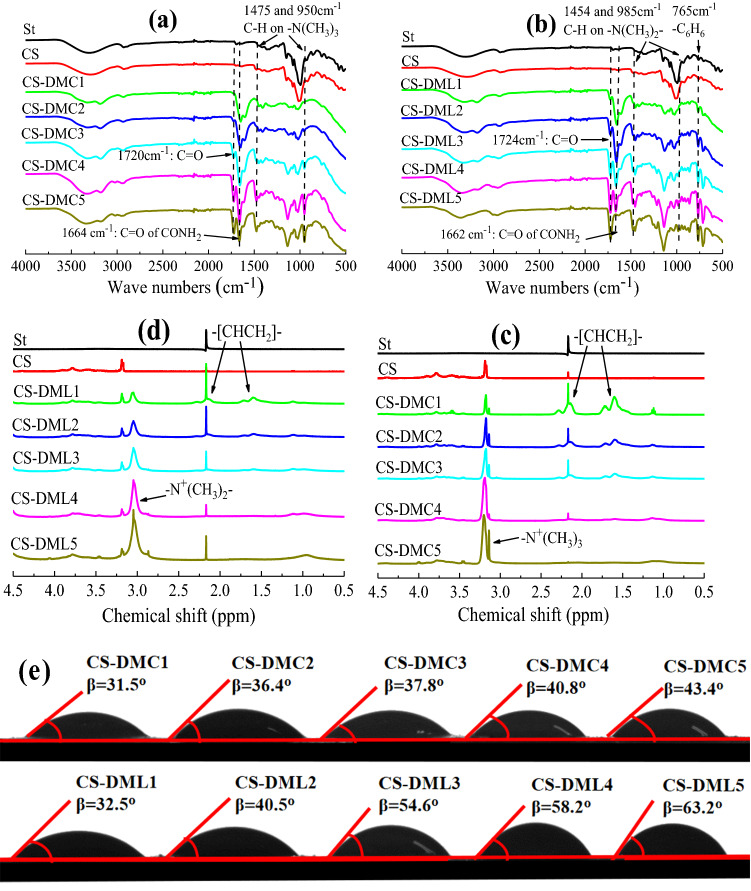
(**a**, **b**) FTIR, (**c**, **d**) ^1^H NMR spectra, and (**e**) contact angle of Starch (St), CS, CS-DMCs and CS-DMLs samples.

CS-DMCs and CS-DMLs were designed and prepared (Scheme 1), and the composition details were presented in experimental section. Their FTIR and ^1^H NMR spectra are shown in Fig.1a–d to characterize the molecular structures of obtained flocculants. The broad peaks at 3200–3600 cm^−1^ was due to the stretching vibration of O–H on the saccharide ring were appeared in all FTIR spectra of St-based samples (Fig.1a–b). After graft copolymerization, the new appeared characteristic peak at around 1662–1664 cm^−1^ assigned to the stretching vibration of C=O on the –CONH_2_ of AM^34^, while that at about 1720–1724 cm^−1^ attributed to the stretching vibration of C=O and those at around 1454–1475 and 950–985 cm^−1^ to the bending vibrations of C-H on quaternary ammonium salt groups were all ascribed to the other grafted cationic monomer (DMC or DML) (Fig.1a,b)^35^. Moreover, the distinct characteristic peak at 765 cm^−1^ in the FTIR spectra of CS-DMLs was due to the bending vibration of C–H on the aromatic skeleton of DML monomer (Fig. 1b)^20,36^. As for their ^1^H NMR spectra (Fig.1c,d), the chemical shift at about 3.0–3.2 ppm was assigned to the methyl proton on the grafted quaternary ammonium salt monomer (DMC or DML)^12,35,37^. The signals of methylene and methine protons on AM monomer were observed at around 2.2 and 1.6 ppm, respectively^30^. The FTIR and ^1^H NMR spectra further confirmed CS-DMCs and CS-DMLs were successfully obtained.

Meanwhile, the CD and hydrophilicity of various St-based flocculants were detected, which are listed in Table 1 and Fig. 1e. With the increase in the amount of the fed cationic monomer (DMC and DML), the CD and contact angle of CS-DMCs and CS-DMLs were increased (Table 1), indicating the positive charge of these flocculants was enhanced but their hydrophilicity were reduced due to the simultaneously increased hydrophobic groups on the quaternary ammonium salts. Besides, the five CS-DMCs (CS-DMC1–CS-DMC5) and the five CS-DMLs (CS-DML1–CS-DML5) designed had similar CD to one another but different hydrophilicity for better comparison, and CS-DML exhibited more hydrophobicity than corresponding CS-DMC because of higher hydrophobic aromatic groups existed on the DML monomer (Table 1 and Fig. 1e). In addition, since the molar ratio of CS to the sum of two monomers was kept a constant about 1:7, the mass ratio of APS to CS was always approximately 1:10 and the yields of final products were more than 90% in all reactions, the average number of graft-chain per saccharide ring and the average number of grafted monomers per branched chain of obtained CS-DMCs and CS-DMLs were roughly estimated and similar to one another about 0.005 and 1400 on the basis of our previously reported method^26,38^. Therefore, the effects of grafted-chain distributions on the dewaterability of these prepared flocculants were minor and were ignored in this work.

**Table 1:** The structural parameters of various St-based flocculants and its dewatering performance.

Samples	Charge density (mmol/g)	Contact angle (°)	Optimal dose (mg/g TSS)	FCMC (%)	SRF (× 10^12^ m/kg)	Bound water content (g/g TSS)	Coefficient of compressibility
CS-DMC1	0.741	31.5	30.0	87.73 ± 0.50	3.765 ± 0.128	2.43 ± 0.12	0.914 ± 0.024
CS-DMC2	1.283	36.4	26.0	85.48 ± 0.13	2.099 ± 0.109	2.03 ± 0.16	0.906 ± 0.019
CS-DMC3	1.895	37.8	18.0	83.35 ± 0.14	1.908 ± 0.108	1.87 ± 0.17	0.884 ± 0.014
CS-DMC4	2.793	40.8	14.0	81.42 ± 0.32	1.928 ± 0.205	1.74 ± 0.11	0.863 ± 0.011
CS-DMC5	3.306	43.4	10.0	81.65 ± 0.50	1.714 ± 0.139	1.73 ± 0.08	0.857 ± 0.009
CS-DML1	0.727	32.5	30.0	86.51 ± 0.15	3.175 ± 0.098	2.33 ± 0.21	0.871 ± 0.015
CS-DML2	1.254	40.5	22.0	85.01 ± 0.26	1.931 ± 0.121	1.83 ± 0.08	0.846 ± 0.008
CS-DML3	1.924	54.6	14.0	82.72 ± 0.30	1.682 ± 0.172	1.79 ± 0.12	0.812 ± 0.010
CS-DML4	2.736	58.2	10.0	80.14 ± 0.48	1.506 ± 0.038	1.67 ± 0.14	0.798 ± 0.007
CS-DML5	3.249	63.2	7.5	80.24 ± 0.49	1.576 ± 0.071	1.61 ± 0.11	0.792 ± 0.008

### Effects of flocculants’ CD and hydrophilicity on sludge dewatering

#### FCMC and SRF

**Figure 2:**
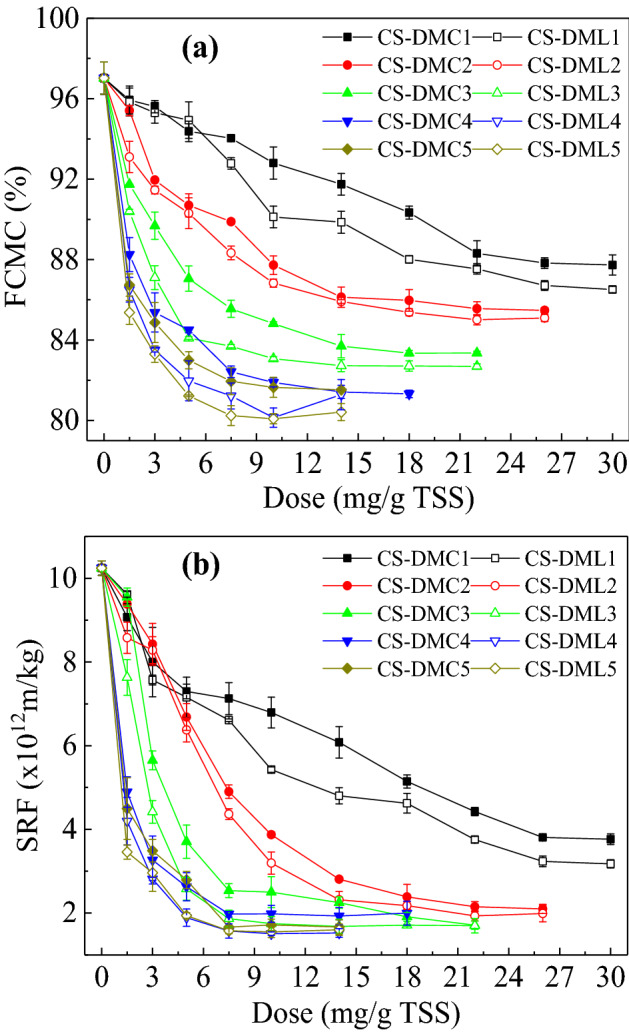
Dose effects of CS-DMCs and CS-DMLs on sludge dewatering performance: (**a**) FCMC and (**b**) SRF.

Figure 2 showed the FCMC and SRF of sludge conditioned by using CS-DMCs and CS-DMLs with different CD and hydrophilicity. Most of FCMC and SRF of sludge rapidly decreased and then gradually achieved a platform with the flocculants dose increasing, indicating efficient dewaterability of these St-based flocculants. The dewatering performance of CS-DMCs and CS-DMLs was improved with the increase of the CD. Besides, the contents of bound water in sludge cakes also decreased as the increase of the two St-based flocculants’ CD (Table 1). CS-DMC5 and CS-DML5 with the highest CD exhibited the lowest FCMC, SRF and bound water contents but the least optimal doses in respective series of St-based flocculants (Fig. 2 and Table 1). The St-based flocculants containing more cationic groups enhanced their charge neutralization for more effective agglomeration of negatively charged sludge particles^9,20^. Besides, the polymeric flocculants with higher CD caused more extended chain conformation in solution and thus improved bridging flocculation effects^39–41^. These two effects caused the sludge dewaterability of these St-based flocculants was positively proportional to their CD, which results are exactly the same as previous reports^40^. In addition, the zeta potentials of sludge supernatants and relevant dewatering performance by adding various flocculants’ doses are displayed in Supporting Information Fig. S1. The zeta potentials were rising rapidly with the increase in flocculants’ dose due to the charge neutralization effect^37,42^. In addition, the zeta potentials were all near to zero under the optimal dose, i.e. reaching to the lowest FCMC and SRF, which illuminated full charge neutralizations were obtained and flocculation of sludge by these St-based flocculants mainly obeyed the simple charge neutralization not patching mechanism^37,42^.

Furthermore, CS-DML showed lower FCMC, SRF and bound water contents than CS-DMC under similar CD to one another, illuminating CS-DML had better sludge dewatering due to its enhanced hydrophobicity association effect^14,20,36^. The incorporated aromatic groups notably improved hydrophobicity of CS-DML, and were beneficial to formation of hydrophobic region and construction of internal water drainage channel.

#### Characteristics of sludge flocs and cakes

**Figure 3:**
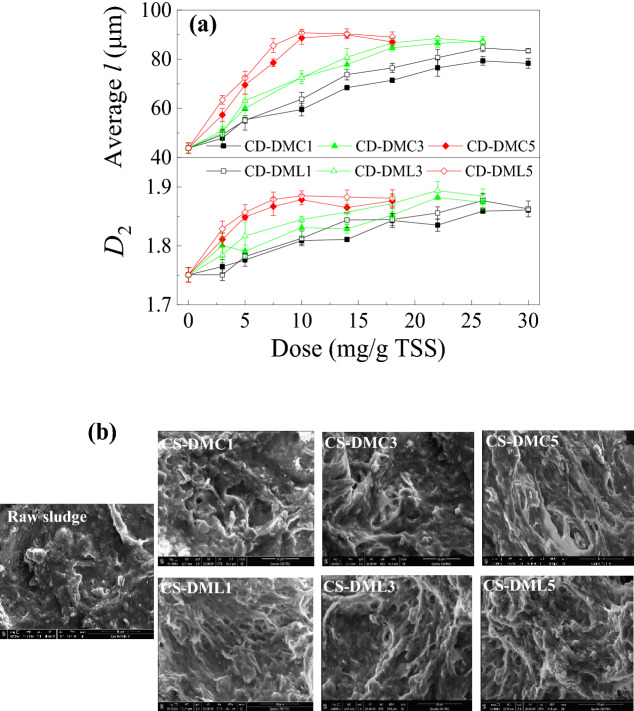
The effects of flocculation conditioning on (**a**) sludge floc size and *D*_2_, and (**b**) surface morphologies of sludge cakes.

The size and *D*_2_ of sludge flocs conditioned by various flocculants are shown in Fig. 3a. The changes in the size and *D*_2_ of sludge flocs with flocculant dose were almost parallel to those in FCMC and SRF (Fig. 2). The size and *D*_2_ increased as the flocculant dose rose, and achieved maximal values at optimal doses (Fig. 3a). These flocculants with higher CD obtained larger and denser sludge flocs, which attributed to enhance charge neutralization and adsorption bridging effects^43^. Moreover, the size and *D*_2_ of sludge flocs obtained by each CS-DML were generally higher than those by the corresponding CS-DMC with the similar CD because of the promoted hydrophobic association effect of CS-DML^14,36^. The aromatic groups on CS-DML can more effectively bind with hydrophobic fragments of sludge except for the electrostatic interactions, the distinct amphiphilic structure of CS-DML was thus resultant in better sludge floc properties and improved dewaterability^14,20,36^. Besides, CS-DMC and CS-DML were graft St-based flocculants and exhibited larger sludge size but less *D*_2_ than the linear St-based one with the similar CD^9^. The grafted-chain architecture due to its more extended chain confirmation may cause enhanced bridging effect and obtain larger floc size but the steric hindrance effects of the branched chains would restrict the internal closeness of the sludge flocs and result in relatively looser floc structure.

SEM micrographs of resultant sludge cakes conditioned by various flocculants at their optimal doses are shown in Fig. 3b. In comparison to the sludge cake without conditioning exhibited a relatively flat and smooth surface, those after conditioning contained evident channels and voids. Besides, the surface of sludge cakes conditioned by CS-DMLs exhibited more notable drainage channels and net-like porosity structures, which were attributed to the enhanced hydrophobic association and formation of more hydrophobic regions in sludge cakes except for charge neutralization and bridging effects^14,36,43^. Moreover, these porous structures were obviously beneficial to improve sludge filterability and compressibility^44,45^. The compressibility coefficients of sludge cakes conditioned at optimal flocculants’ doses are all listed in Table 1. The *S* of sludge after conditioning was obviously lower than that without conditioning approximately 1.195. The *S* of sludge were decreased with the increase in the flocculants’ CD (Table 1) and each CS-DML had a lower *S* than the corresponding CS-DMC with the similar CD, indicating improvement of CD and hydrophobicity in those St-based flocculants can cause the filter cakes more permeable and acquire better sludge filterability and dewaterability^44,45^. Those findings were ascribed to the improved charge neutralization and bridging effects and enhanced hydrophobic association^14,36^. The results of sludge flocs and cakes by the various flocculants were quite consistent with those of FCMC and SRF (Fig. 2).

#### EPS analysis

**Figure 4:**
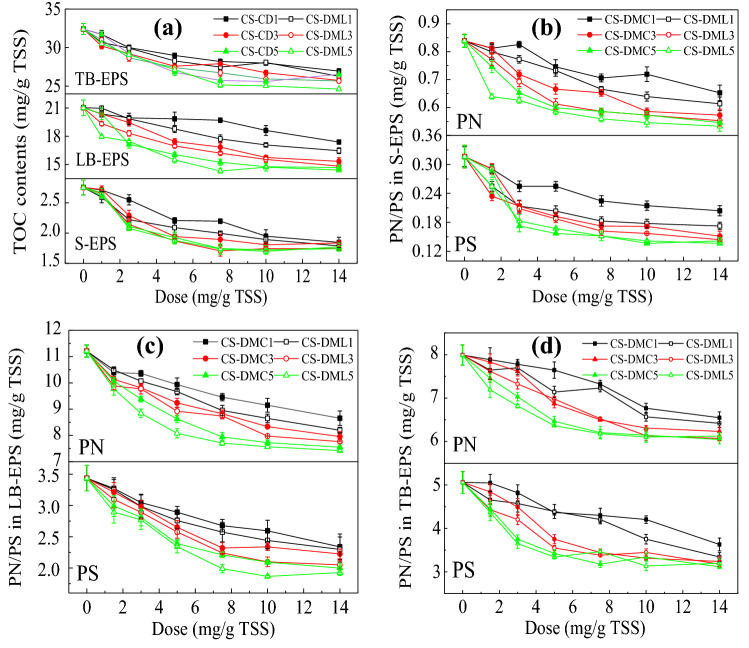
(**a**) TOC content in each of the EPS, PN and PS content of (**b**) S-EPS, (**c**) LB-EPS, and (**d**) TB-EPS in sludge treated by various St-based flocculants.

EPS is an important component in sludge to influence the sludge dewaterability^46,47^. The TOC contents after conditioned by various St-based flocculants are shown in Fig. 4a. Similarly to the change trends of FCMC and SRF (Fig. 2), the TOC contents of S-EPS, LB-EPS, and TB-EPS all decreased with the flocculants’ doses increasing until the lowest values were achieved at their optimal doses. The St-based flocculants can effectively compress the three fractions of EPS through strong charge neutralization and bridging effects^9,23^. Thus, S-EPS was aggregated and precipitated while LB-EPS was mainly transformed into TB-EPS, causing the decrease in the TOC contents of S-EPS and LB-EPS. However, the decrease in the apparent TOC content of TB-EPS may be due to the fact that many of TB-EPS might bind to the sludge particles too tightly after effective coagulation and flocculation to be detected by currently employed measurement^3,46^. Besides, according to Fig. 4a, these flocculants with higher CD generally exhibited less residual TOC contents at the same doses because of enhanced charge neutralization and bridging effects^9,23^, and CS-DML usually caused a higher efficiency in removal of EPS than corresponding CS-CD with the similar CD due to improved hydrophobic association^14,20^. Moreover, CS-DML5 had excellent EPS compression effects, by which the residual content of each EPS fraction was lower than that by CS-DMC5 and some traditional coagulants and flocculants including FeCl_3_ and CPAM (Supporting Information Table S1). The enhanced CD and hydrophobicity caused more efficient to compress and reduce EPS and thus higher sludge dewatering performance^14,20,36^.

More specifically, the contents of PN and PS have been tested and presented in Fig.4b–d. According to Fig. 4, the PN and PS contents in three EPS fractions showed similar flocculant doses, CD, and hydrophobicity dependences to the TOC contents after conditioned by these flocculants. CS-DML5 with a higher CD and a stronger hydrophobicity exhibited more evident compressions of PN and PS. The hydrophobic groups of the St-based flocculants can efficiently bind to the hydrophobic segments of PN and PS besides electrostatic interactions. The original gel-like suspended structures of sludge were thus destroyed substantially and the bounded water was released.

**Figure 5:**
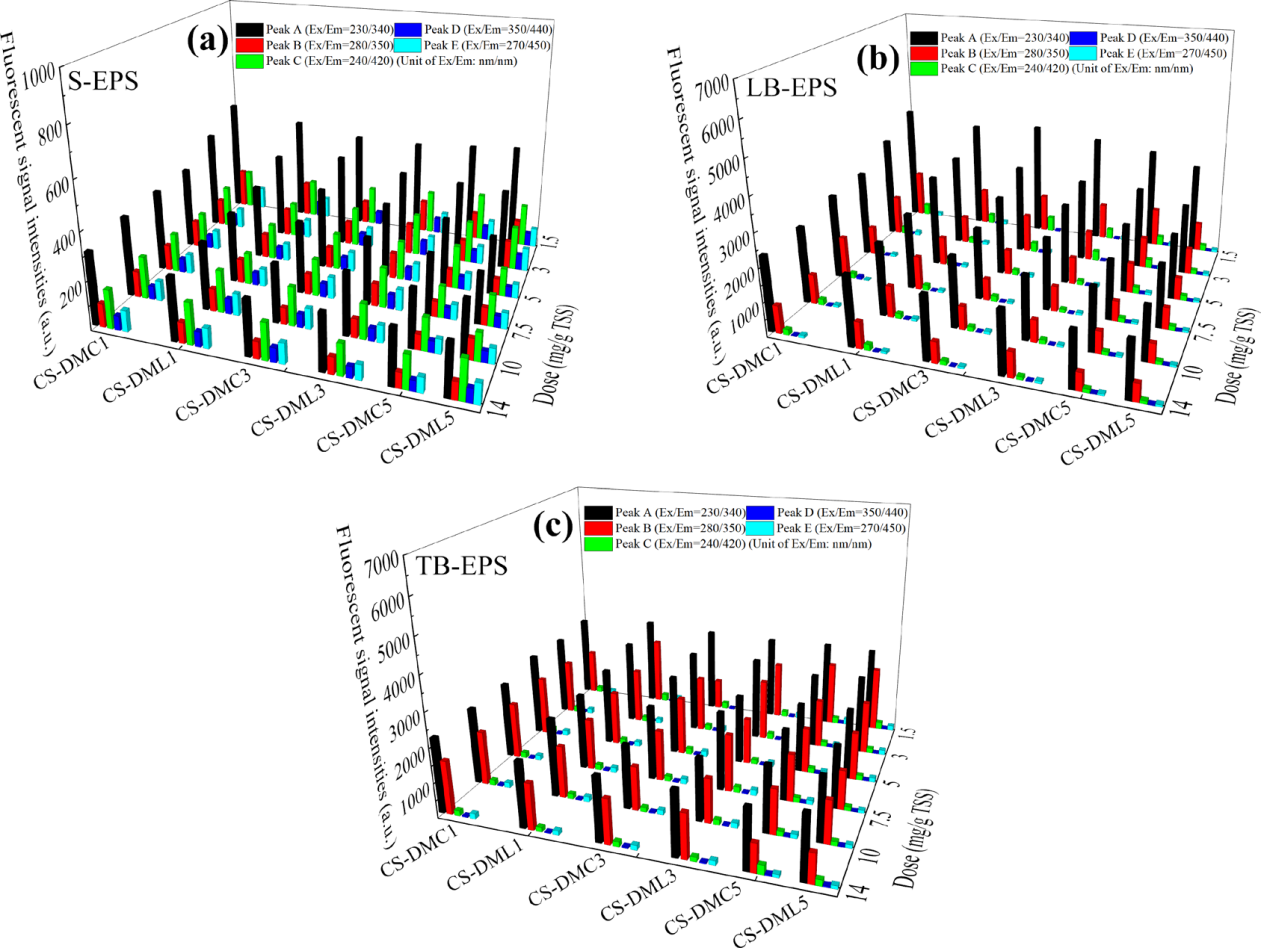
The intensities of various 3D-EEM characteristic peaks of (**a**) S-EPS, (**b**) LB-EPS, and (**c**) TB-EPS in sludge treated by the St-based flocculants.

3D-EEM spectroscopy was used to further characterize the changes of fluorescent components in three EPS fractions. Based on previous reports, the five fluorescent peaks were extracted and represented 5 types of substances including aromatic PN (λ_ex/em_ = 230 nm/340 nm, Peak A), tryptophan PN (λ_ex/em_ = 280 nm/350 nm, Peak B), fulvic acid (λ_ex/em_ = 240 nm/420 nm, Peak C), and humic substances (λ_ex/em_ = 350 nm/440 nm and 270 nm/450 nm for Peaks D and E, respectively)^48,49^. Supporting Information Figs. S2–S5 showed EEM profiles of three EPS fractions before and after conditioned by various flocculants, and their intensities of the five fluorescent peaks are accordingly shown in Fig. 5. Based on Fig. 5, the intensities of Peaks A and B in three EPS fractions were weakened after conditioning, which was consistent with the changes of PN contents (Fig.4b–d), but no evident changes in the intensities of Peaks C, D and E were observed, indicating the contents of the corresponding fulvic acid and humic substances could not be affected by the St-based flocculants and thus had no direct relationship with the sludge dewaterability. These results were fully consistent with previous reports that sludge dewaterability was closely related to the content of PN substances but not humic substances and low PN content in EPS had a better dewatering performance^50^. Furthermore, CS-DML5 was more effective in compression of Peaks A and B due to that the aromatic groups contained had stronger interactions with the hydrophobic groups on aromatic PN.

In short, the contents of total EPS including three fractions and their two main components were significantly reduced after CS-DMCs and CS-DMLs conditioning, and increases in CD and hydrophobicity of the St-based flocculants caused more efficient compression effects and better sludge dewaterability.

## Modeling

### Modeling of structural factors

To quantitatively analyze the effects of CD and hydrophobicity on dewatering performance of these flocculants, a second-order polynomial model was introduced on the basis of phenomenological theory to reveal the relationship between flocculants’ structure and dewatering^26,33^, as shown in Eq. (1). The independent variables, total charge content (CC) and hydrophobic coefficient (θ) were functions of the FCMC (Y). The detailed model analysis process was presented in the Section of Method.

1 $${\text{Y}} = b_{0} + b_{{{\text{CC}}}} \cdot {\text{CC}} + \, b_{\theta } \cdot \theta + b_{{{\text{CC}} \times \theta }} \cdot {\text{CC}} \cdot \theta + \, b_{{{\text{CC}}}}^{2} \cdot {\text{CC}}^{2} + b_{\theta }^{2} \cdot \theta^{2 } ({\text{CC }} = {\text{ CD}} \cdot {\text{dose}})$$

2 $${\text{Y}}_{(CS - DMCs)} = 106.0 - 53.52{\text{CC}} - 3.257\theta + \, 94.12{\text{CC}} \cdot \theta + 34.32{\text{CC}}^{2} - 286.9\theta^{2} \left( {R^{2} = 0.9615} \right)$$

3 $${\text{Y}}_{(CS - DMLs)} = 99.58 - 46.93{\text{CC }} - 8.522\theta + \, 22.48{\text{CC}} \cdot \theta + 50.97{\text{CC}}^{2} - 72.25\theta^{2} \left( {R^{2} = 0.9649} \right)$$

**Figure 6:**
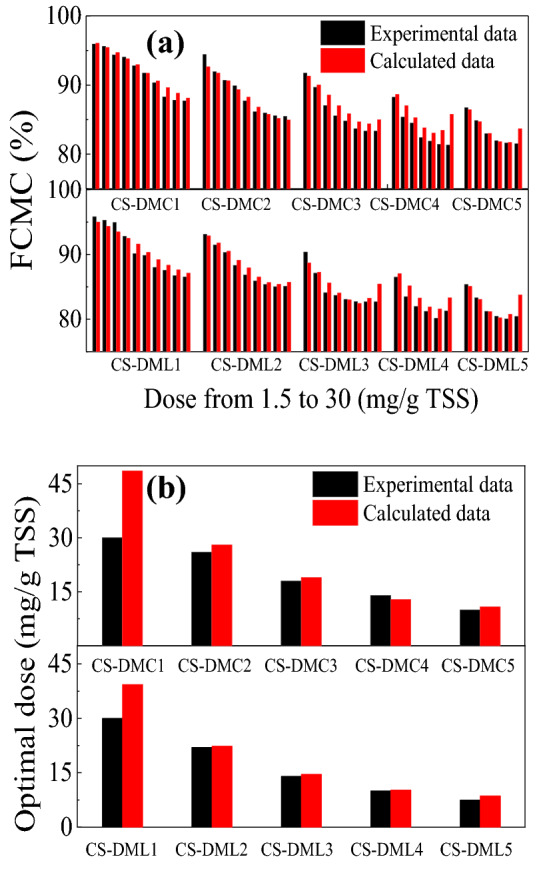
Comparison of the experimental and calculated (**a**) FCMC and (**b**) optimal doses of various St-based flocculants based on Eqs. (1)–(6) in the sludge dewatering.

Equation (1) was applied to simulate the dewatering curves of CS-DMCs and CS-DMLs (Fig. 2a), and the obtained fitting coefficients were showed in Eqs. (2) and (3), respectively. The actual experimental and simulated FCMC data were presented and compared in Fig. 6a. Those FCMC data was primarily consistent to one another and the correlation coefficients (*R*^2^) were both greater than 0.96. Therefore, the second-order polynomial model could effectively describe the sludge dewatering performance.

According to Eqs. (2) and (3), the linear coefficients of *b*_*CC*_ and *b*_θ_ for these flocculants were all negative, which confirmed that improving CD and hydrophobicity can further reduce the FCMC and enhance the sludge dewaterability. Moreover, the absolute value of *b*_θ_ for CS-DMC approximately 3.257 was much lower than that for CS-DML about 8.522, demonstrating the hydrophobic association of CS-DML was more notable in sludge dewatering. These theoretic finding were fully consistent with previously experimental results (Figs. 2, 3, 4, 5) that the St-based flocculants with higher CC and stronger hydrophobicity showed better sludge dewatering performance due to the synergistic effects of charge neutralization, bridging flocculation and hydrophobic association^3,14,36^.

### Theoretical estimation

The required dose was not only a critical factor influencing the application cost of flocculants but also a substantial one on sludge dewaterability. Given the notable contribution of CD to the sludge dewatering performance and the optimal dose (*dose*_*opt*_), Eq. (4) was achieved by differentiating Eq. (1) in regard to dose, as shown below,

4 $$dose_{opt} = - \frac{{b_{cc} + b_{cc \times \theta } \cdot \theta }}{{2bcc^{2} \cdot CD}}$$

5 $$dose_{{opt(CS - DMCs)}} = - \frac{{b_{{cc}} + b_{{cc \times \theta }} \cdot \theta }}{{2bcc^{2} \cdot CD}} = - \frac{{ - 53.52 \cdot + 94.12\theta }}{{2 \cdot 34.32CD}}$$

6 $$dose_{{opt(CS - DMLs)}} = - \frac{{b_{{cc}} + b_{{cc \times \theta }} \cdot \theta }}{{2bcc^{2} \cdot CD}} = - \frac{{ - 46.93 + 22.48\theta }}{{2 \cdot 50.97CD}}$$

By combination of Eqs. (2) and (3), the theoretic optimal doses of CS-DMC and CS-DML in sludge conditioning can be estimated according to Eqs. (5) and (6) on the basis of given CD and *θ*. The calculated and experimental optimal doses were listed and compared in Fig. 6b. The calculated optimal doses were approximately the same as the experimental ones, further confirming the good applicability of this model. However, the theoretically predicted results had relatively large deviations in CS-DMC1 and CS-DML1 due to their lower CD contained. Other effects associated with their structural characteristics, such as hydrophobic association and bridging flocculation effects, had more notable contributions to the sludge dewatering except for charge neutralization. In summary, the dewatering properties and optimal doses of these flocculants could be predicted by establishing structure–activity relationship [Eqs. (1)–(6)].

## Conclusion

In this work, the effects of CD and hydrophobicity of two graft cationic St-based flocculants, CS-DMCs and CS-DMLs, on the sludge dewatering were evaluated and compared from both experimental and theoretic aspects. The experimental findings was approximately the same as the theoretic results. The sludge filterability and compressibility were enhanced with the increase of the CD and hydrophobicity of these St-based flocculants, because of the synergistic effects of charge neutralization, bridging flocculation and hydrophobic association. CS-DML with a higher CD and a stronger hydrophobicity resulted in lower FCMC, SRF and bound water content, larger and denser sludge flocs, lower *S* of sludge cakes, more efficient in EPS compression, and less optimal doses. In addition to electrostatic interactions, the hydrophobic groups on flocculants can more effectively bind to the hydrophobic segments of sludge, causing the destruction of the original hydrated gel-like structure, formation of more drainage channels and net-like porosity in sludge cake, and efficient release of bound water. On the basis of the employed second-order polynomial model, the structure–activity relationship of these St-based flocculants in sludge dewatering was well established, accordingly, the sludge dewatering and the optimal dose of flocculants can be estimated successfully. Although more practices were still needed, the hydrophobically associating cationic starch-based flocculants with amphipathic structure were confirmed to have superior sludge dewatering performance. This work thus provided a novel and valuable way to exploit and design of low-cost and high-performance natural polymeric conditioners.

## Experiment

### Reagents

St, weight-average molecular weight of approximately 1.5 × 10^5^ g/mol, was purchased from Binzhou Jinhui Corn Development Co., Ltd. DML (60wt% in water) was procured from Nanjing Bangnuo Bilogical Technological Co., Ltd. DMC was supplied by Shanghai Bangcheng Bilogical Technological Co., Ltd. AM, anthrone, bovine serum albumin, and toluidine blue O were purchased from Sinopharm Chemical Reagent Co., Ltd. 3-Chloro-2-hydroxypropyltrimethyl ammonium chloride (CTA, 60wt% in water), glucose, and poly(vinyl sulfate) potassium salt were obtained from Aladdin Industrial Co. Sodium chloride, Ferric chloride, sodium hydrate, and ammonium persulfate (APS) were from Shanghai Lingfeng Chemical Reagent Co., Ltd. Cationic polyacrylamide (CPAM), with the weight-average molecular weight of approximately 1.0 × 10^7^ g/mol and CD of 1.06 ± 0.13 mmol/g, was purchased from Dongying Nuoer Chemical Co., Ltd. The reagents used here were of analytical grade, except where noted.

The sludge was taken from a municipal sewage treatment plant in Nanjing, China. Sludge was placed at 4 °C in the refrigerator and tested within 7 days. The moisture content was approximately 98.5%, and the ratio of volatile suspended solids (VSS) to total suspended solids (TSS, VSS/TSS) was 48.6 ~ 56.4%^51^. The physicochemical characteristics of raw sludge and the detailed measurements were presented in Supporting Information Table S2.

### Preparation and characterization of the St-based flocculants

On the basis of our last report^26^, St was first slightly etherified using CTA to enhance its water solubility, and the final product was called CS. The CS with a CD of 0.643 mmol/g was applied for the following graft copolymerization. Briefly, the initiator APS, with the mass ratio of APS:CS approximately 1:10, was rapidly added into an aqueous solution containing 5.0 g of pre-dissolved CS with a keep stirring under nitrogen environment at 55 °C. After 10.0 min, two monomers, i.e., DML and AM or DMC and AM, mixed in a homogeneous aqueous solution with a designed molar ratio, were slowly added to the above mixture drop by drop. After 3 h reaction, the final samples were precipitated, dried, crushed, and purified. The two series of CS-DMCs and CS-DMLs with different components were prepared by feeding monomers with different mole ratios. However, the molar ratio of CS to the sum of two monomers was kept a constant about 1:7. CS-DMCs and CS-DMLs Scheme 1 shows the different structural morphologies and the synthesis processes of the two St-based flocculants.

CS-DMCs and CS-DMLs were basically characterized by ^1^H NMR at 400 MHz using D_2_O as the solvent (Bruker Ascend 400) and FTIR (Bruker Model IFS 66/S). The CDs of the St-based flocculants were tested by using a colloid titration method and their hydrophobicity was by the determination of a contact angle measurement system (DSA100, Kruss, Germany)^23^, both of which are listed in Table 1.

### Sludge conditioning

The stock aqueous solution of flocculants was freshly prepared with the concentration of 3.0 g/L for the following dewatering experiments. The raw sludge was flocculated by adding a known amount of St-based flocculant by using a program-controlled paddle apparatus (Model TA6, Wuhan Hengling Technology Co., Ltd.). The flocculated procedure was a rapid stirring for 1.0 min at 250 rpm, and then followed by a slow stirring for 5.0 min at 50 rpm. After standing still for another 30.0 min, the conditioned sludge was used for the following analysis of dewatering performance.

### Analytical methods

#### FCMC, SRF and compressibility

The sludge dewaterability of the flocculants was investigated by analyzing FCMC, in the light of the standard method^51^. SRF, another popular indicator evaluating the dewatering performance, was determined as follows, the conditioned sludge was filtered using a Buchner funnel with 0.45 µm membrane at 0.05 MPa pressure. The entire process of filtering is recorded before terminating the filtering after 7.0 min or breaking the vacuum state. The volume of filtrate can obtain from the video. The dewatering equipment was schematically described in Supporting Information Fig. S6. SRF was further estimated based on Eq. (7):

7 $$SRF = \frac{{2PA^{2} b}}{\mu \omega }$$

where *P* (kg/m^2^) is pressure; *A* (m^2^) denotes the filter area; *b* (s/m^6^) is the slope of filtrate discharge curve; *μ* (kg s/m^2^) is for kinetic viscosity; and *ω* (kg/m^3^) represents dry solid weight per unit volume sludge on the filtrate media.

The coefficient of compressibility (*S*), indicating the compressibility of sludge cake, was evaluated and quantified according to Eq. (8):

8 $$\frac{{SRF_{1} }}{{SRF_{2} }} = \left( {\frac{{P_{1} }}{{P_{2} }}} \right)^{S}$$

where *SRF*_1_ and *SRF*_2_ are the *SRF*s under two distinct pressures *P*_1_ (0.03 MPa) and *P*_*2*_ (0.05 MPa), respectively.

#### Characterization of sludge floc properties

The sludge flocs were photographed by a Pentax Model K-m digital camera with a 200-mm len under a fixed magnification, and the characteristic floc length (*l*) and its projected area (*A*) were obtained by using an image analysis software (Image-Pro Plus 6.0)^52,53^. The two-dimensional fractal dimensions (*D*_2_) of flocs was calculated as the slope of a plot of *log A* versus *log l* based on Eq. (9):

9 $${\text{A}} \propto {\text{l}}^{{{\text{D}}_{{2}} }}$$

Besides, a scanning electron microscope (SEM, FEI Quanta 250) was used to directly observe the surface morphologies of sludge flocs obtained by freeze-drying pretreatment. The content of bound water in sludge cake was measured by a differential scanning calorimetry analyzer (TA, US), and the detailed detected method was described in Supporting Information Text S1. The zeta potentials of sludge supernatant containing the colloids of suspended flocs were measured by a Zetasizer Nano Z (Malvern, UK).

#### Extraction and analysis of EPS

An extraction method was used to separate soluble EPS (S-EPS), loosely bound EPS (LB-EPS), and tightly bound EPS (TB-EPS) from sludge according to the previous report^54^, and the detailed extraction process was presented in Supporting Information Text S2. The three EPS fraction was filtered through a 0.45 µm membrane to remove solid sludge impurities before analysis of dissolved organic matter. Total organic carbon (TOC) was measured by using multi-N/C 3100 TOC (Jena, German). Protein (PN) concentration in each EPS fraction was measured using coomassie brilliant blue G-250 method with BSA as standard and polysaccharide (PS) was determined by the anthrone method with glucose as standard both through a UV-2600A spectrometer (Unico, US)^55,56^. Three-dimensional excitation emission matrix (3D-EEM) spectra were performed on a F-7000 fluorescence spectrophotometer (Hitachi, Japan) with an excitation range of 200–450 nm at 5 nm increments and an emission range of 250–550 nm at 1 nm increment. The voltage of the photomultiplier tube was 700 V^23^. All experiments were tested in triplicate, and the average of replicates would be a final result with the error bars on behalf of standard deviation.

### Model analysis

A second-order polynomial model was introduced on the basis of phenomenological theory to reveal the relationship between flocculants’ structure and dewatering^26,33^, as shown in Eq. (10). The hydrophobic coefficient of the St-based flocculants (*θ*) was defined as the quotient of contact angle (*β*) to straight angle (180°) as shown in Eq. (11). The two independent structural variables, CD and *θ*, were thus used. Given that the actual effects of charge neutralization in sludge dewatering should be ascribed to the total charge content (CC) not CD, CC, equal to the product of CD and flocculant dose, was employed instead of CD, as showed in Eqs. (12) and (13).

10 $$Y = b_{0} + \sum\limits_{i = 1}^{n} {b_{i} x_{i} } + \sum\limits_{i = 1}^{n} {b_{ii} x_{i}^{2} } + \sum\limits_{i = 1}^{n - 1} {\sum\limits_{j = i + 1}^{n} {b_{ij} x_{i} x_{j} } }$$

11 $$\theta { = }\frac{\beta }{180}$$

12 $${\text{Y }} = b_{0} + b_{{{\text{CC}}}} \cdot{\text{CD}}\cdot{\text{dose}} + \, b_{\theta } \cdot\theta + b_{{{\text{CC}} \times \theta }} \cdot{\text{CD}}\cdot{\text{dose}}\cdot\theta \, + b_{{{\text{CC}}}}^{{2}} \cdot{\text{CD}}^{{2}} \cdot{\text{dose}}^{{2}} + b_{\theta }^{{2}} \cdot\theta^{{2}}$$

13 $${\text{Y}} = b_{0} + b_{{{\text{CC}}}} \cdot{\text{CC}} + \, b_{\theta } \cdot\theta + b_{{{\text{CC}} \times \theta }} \cdot{\text{CC}}\cdot\theta + \, b_{{{\text{CC}}}}^{{2}} \cdot{\text{CC}}^{{2}} + b_{\theta }^{{2}} \cdot\theta^{{{2} }} ({\text{CC }} = {\text{ CD}}\cdot{\text{dose}})$$

where Y refers to the FCMC; *b*_*0*_ stands for the constant coefficient; *b*_*CC*_ and *b*_*θ*_ are the linear coefficients corresponding to CC and *θ* respectively; and *b*_*CC*×*θ*_, *b*_*CC*_^2^ and *b*_*θ*_^2^ are the quadratic coefficients.

## Supplementary Information

Supplementary Information.

## References

[1] Christensen ML, Keiding K, Nielsen PH, Jorgensen MK (2015). Dewatering in biological wastewater treatment: A review. Water Res..

[2] Luo J, Zhang Q, Zha J, Wu Y, Wu L, Li H, Tang M, Sun Y, Guo W, Feng Q, Cao J, Wang D (2020). Potential influences of exogenous pollutants occurred in waste activated sludge on anaerobic digestion: A review. J. Hazard Mater..

[3] Wei H, Gao BQ, Ren J, Li AM, Yang H (2018). Coagulation/flocculation in dewatering of sludge: A review. Water Res..

[4] Long GY, Zhu PT, Shen Y, Tong MP (2009). Influence of extracellular polymeric substances (EPS) on deposition kinetics of bacteria. Environ. Sci. Technol..

[5] Wingender J, Neu TR, Flemming HC (1999). Microbial Extracellular Polymeric Substances: Characterization, Structure and Function.

[6] Cai MQ, Hu JQ, Wells G, Seo Y, Spinney R, Ho SH, Dionysiou DD, Su J, Xiao RY, Wei ZS (2018). Understanding mechanisms of synergy between acidification and ultrasound treatments for activated sludge dewatering: From bench to pilot-scale investigation. Environ. Sci. Technol..

[7] Kim MS, Lee KM, Kim HE, Lee HJ, Lee C, Lee C (2016). Disintegration of waste activated sludge by thermally-activated persulfates for enhanced dewaterability. Environ. Sci. Technol..

[8] Lapointe M, Barbeau B (2017). Dual starch-polyacrylamide polymer system for improved flocculation. Water Res..

[9] Lv S, Sun T, Zhou Q, Liu J, Ding H (2014). Synthesis of starch-g-p(DMDAAC) using HRP initiation and the correlation of its structure and sludge dewaterability. Carbohyd. Polym..

[10] Jorand F, Boue-Bigne F, Block JC, Urbain V (1998). Hydrophobic/hydrophilic properties of activated sludge exopolymeric substances. Water Sci. Technol..

[11] Wei L, Xia X, Zhu F, Li Q, Xue M, Li J, Sun B, Jiang J, Zhao Q (2020). Dewatering efficiency of sewage sludge during Fe^2+^ activated persulfate oxidation: Effect of hydrophobic hydrophilic properties of sludge EPS. Water Res..

[12] He DQ, Wang LF, Jiang H, Yu HQ (2015). A Fenton-like process for the enhanced activated sludge dewatering. Chem. Eng. J..

[13] Liu XM, Sheng GP, Luo HW, Zhang F, Yuan SJ, Xu J, Zeng RJ, Wu JG, Yu HQ (2010). Contribution of extracellular polymeric substances (EPS) to the sludge aggregation. Environ. Sci. Technol..

[14] Zhou Y, Zheng H, Wang Y, Zhao R, Liu H, Ding W, An Y (2020). Enhanced municipal sludge dewaterability using an amphiphilic microblocked cationic polyacrylamide synthesized through ultrasonic-initiation: Copolymerization and flocculation mechanisms. Colloid. Surf. A..

[15] Lee CS, Robinson J, Chong MF (2014). A review on application of flocculants in wastewater treatment. Process Saf. Environ..

[16] Shon HK, Vigneswaran S, Kim IS, Cho J, Kim GJ, Kim JB, Kim JH (2007). Preparation of titanium dioxide (TiO_2_) from sludge produced by titanium tetrachloride (TiCl_4_) flocculation of wastewater. Environ. Sci. Technol..

[17] Sievers M, Schroeder C, Bormann H, Onyeche TI, Schlaefer O, Schaefer S (2003). Automation in sludge dewatering by novel on-line characterisation of flocculation. Water Sci. Technol..

[18] Lee KE, Morad N, Teng TT, Poh BT (2012). Development, characterization and the application of hybrid materials in coagulation/flocculation of wastewater: A review. Chem. Eng. J..

[19] Othmani B, Rasteiro MG, Khadhraoui M (2020). Toward green technology: a review on some efficient model plant-based coagulants/flocculants for freshwater and wastewater remediation. Clean Technol. Environ..

[20] Liu YZ, Zheng HL, Sun YJ, Ren J, Zheng XY, Sun Q, Jiang SJ, Ding W (2020). Synthesis of novel chitosan-based flocculants with amphiphilic structure and its application in sludge dewatering: Role of hydrophobic groups. J. Clean. Prod..

[21] Luo HJ, Ning XA, Liang XJ, Feng YF, Liu JY (2013). Effects of sawdust-CPAM on textile dyeing sludge dewaterability and filter cake properties. Bioresour. Technol..

[22] Niu M, Zhang W, Wang D, Chen Y, Chen R (2013). Correlation of physicochemical properties and sludge dewaterability under chemical conditioning using inorganic coagulants. Bioresour. Technol..

[23] Wei H, Ren J, Li AM, Yang H (2018). Sludge dewaterability of a starch-based flocculant and its combined usage with ferric chloride. Chem. Eng. J..

[24] Guo KY, Gao BY, Wang WY, Yue QY, Xu X (2019). Evaluation of molecular weight, chain architectures and charge densities of various lignin-based flocculants for dye wastewater treatment. Chemosphere.

[25] Jin XG, Bi L, Lyu T, Chen J, Zhang HG, Pan G (2019). Amphoteric starch-based bicomponent modified soil for mitigation of harmful algal blooms (HABs) with broad salinity tolerance: Flocculation, algal regrowth, and ecological safety. Water Res..

[26] Hu P, Xi Z, Li Y, Li A, Yang H (2020). Evaluation of the structural factors for the flocculation performance of a co-graft cationic starch-based flocculant. Chemosphere.

[27] Song YB, Zhang J, Gan WP, Zhou JP, Zhang LN (2010). Flocculation properties and antimicrobial activities of quaternized celluloses synthesized in NaOH/urea aqueous solution. Ind. Eng. Chem. Res..

[28] Thakur MK, Thakur VK, Gupta RK, Pappu A (2016). Synthesis and Applications Of Biodegradable Soy Based Graft Copolymers: A review. ACS Sustain. Chem. Eng..

[29] Thakur VK, Thakur MK (2014). Recent advances in graft copolymerization and applications of chitosan: A review. ACS Sustain. Chem. Eng..

[30] Liu ZZ, Wei H, Li AM, Yang H (2017). Evaluation of structural effects on the flocculation performance of a co-graft starch-based flocculant. Water Res..

[31] Fanta GF, Ceresa RJ (1973). Synthesis of graft and block copolymers of starch. Block and Graft Copolymerization.

[32] Lin Q, Peng H, Zhong S, Xiang J (2015). Synthesis, characterization, and secondary sludge dewatering performance of a novel combined silicon-aluminum-ironstarch flocculant. J. Hazard Mater..

[33] Ginzburg VL, Landau LD (1950). Phenomenological theory. J. Exp. Theor. Phys. USSR.

[34] Song H, Wu D, Zhang RQ, Qiao LY, Zhang SH, Lin S, Ye J (2009). Synthesis and application of amphoteric starch graft polymer. Carbohyd. Polym..

[35] Wang JP, Chen YZ, Yuan SJ, Sheng GP, Yu HQ (2009). Synthesis and characterization of a novel cationic chitosan-based flocculant with a high water-solubility for pulp mill wastewater treatment. Water Res..

[36] Liao Y, Zheng HL, Qian L, Sun YJ, Dai L, Xue WW (2014). UV-initiated polymerization of hydrophobically associating cationic polyacrylamide modified by a surface-active monomer: A comparative study of synthesis, characterization, and sludge dewatering performance. Ind. Eng. Chem. Res..

[37] Yang ZL, Gao BY, Li CX, Yue QY, Liu B (2010). Synthesis and characterization of hydrophobically associating cationic polyacrylamide. Chem. Eng. J..

[38] Hu P, Zhuang SH, Shen SH, Yang YH, Yang H (2021). Dewaterability of sewage sludge conditioned with a graft cationic starch-based flocculant: role of structural characteristics of flocculant. Water Res..

[39] Flory PJ (1953). Principles of Polymer Chemistry.

[40] Guo B, Yu H, Gao BY, Zhang S, Yue QY, Xu X (2017). Novel cationic polyamidine: Synthesis, characterization, and sludge dewatering performance. J. Environ. Sci..

[41] Ren J, Li N, Wei H, Li AM, Yang H (2020). Efficient removal of phosphorus from turbid water using chemical sedimentation by FeCl_3_ in conjunction with a starch-based flocculant. Water Res..

[42] Guibal E, Van Vooren M, Dempsey BA, Roussy J (2006). A review of the use of chitosan for the removal of particulate and dissolved contaminants. Sep. Sci. Technol..

[43] Yang R, Li H, Huang M, Yang H, Li AM (2016). A review on chitosan-based flocculants and their applications in water treatment. Water Res..

[44] Ning X, Luo H, Liang X, Lin M, Liang X (2013). Effects of tannery sludge incineration slag pretreatment on sludge dewaterability. Chem. Eng. J..

[45] Zhao YQ, Bache DH (2001). Conditioning of alum sludge with polymer and gypsum. Colloid. Surf. A..

[46] Sheng GP, Yu HQ, Li XY (2010). Extracellular polymeric substances (EPS) of microbial aggregates in biological wastewater treatment systems: A review. Biotechnol. Adv..

[47] Forster CF (1971). Activated sludge surfaces in relation to the sludge volume index. Water Res..

[48] Jacquin C, Lesage G, Traber J, Pronk W, Heran M (2017). Three-dimensional excitation and emission matrix fluorescence (3D-EEM) for quick and pseudo-quantitative determination of protein and humic-like substances in full-scale membrane bioreactor (MBR). Water Res..

[49] Wei H, Tang YN, Shoeib T, Li AM, Yang H (2019). Evaluating the effects of the preoxidation of H_2_O_2_, NaClO, and KMnO_4_ and reflocculation on the dewaterability of sewage sludge. Chemosphere.

[50] Li XY, Yang SF (2007). Influence of loosely bound extracellular polymeric substances (EPS) on the flocculation, sedimentation and dewaterability of activated sludge. Water Res..

[51] APHA (1998). Standard Methods for the Examination of Water and Wastewater twentiethed.

[52] Chakraborti RK, Atkinson JF, Benschoten JEV (2000). Characterization of alum floc by image analysis. Environ. Sci. Technol..

[53] Chakraborti RK, Gardner KH, Atkinson JF, Benschoten JEV (2003). Changes in fractal dimension during aggregation. Water Res..

[54] Morgan JW, Forster CF, Evison L (1990). A comparative study of the nature of biopolymers extracted from anaerobic and activated sludges. Water Res..

[55] Bradford MM (1976). A rapid and sensitive method for quantitation of microgram quantities of protein utilizing the principle of protein-dye binding. Anal. Biochem..

[56] Frolund B, Palmgren R, Keiding K, Nielsen PH (1996). Extraction of extracellular polymers from activated sludge using a cation exchange resin. Water Res..

