# Supplementary material for: Evaluation of hydrophobically associating cationic starch-based flocculants in sludge dewatering
Source: Sci Rep. 2021 Jun 3;11:11819. doi: 10.1038/s41598-021-91323-y (PMC8175719; doi:10.1038/s41598-021-91323-y)
Supplement: Supplementary file 1 — Supplementary Information. [file 41598_2021_91323_MOESM1_ESM.docx]

**Supporting Information Cover Sheet**

**Evaluation of hydrophobically associating cationic starch-based flocculants in sludge dewatering ***

Pan Hu, Shaohang Shen, Hu Yang^**^

State Key Laboratory of Pollution Control and Resource Reuse, School of the Environment, Nanjing University, Nanjing 210023, P. R. China

Number of pages: 17

Number of table: 2

Number of text: 2

Number of figures: 6

* Supported by the National Natural Science Foundation of China (grant no. 51978325).

** Corresponding author. Tel & Fax: 86-25-89681272, E-mail: yanghu@nju.edu.cn

**Table of Contents**

| **Index** | **Captions** | **Page** |
| --- | --- | --- |
| **Table S1** | The residual TOC contents of various EPS fractions in sludge conditioned by different coagulants and flocculants. | S3 |
| **Table S2** | Characteristics of sludge. | S4 |
| **Text S1** | The measurement methods of bound water content. | S5 |
| **Text S2** | The extraction method of EPS. | S6 |
| **Figure S1** | The zeta potentials and corresponding FCMC and SRF of sludge conditioned by (a) CS-DMC1, (b) CS-DML1, (c) CS-DMC3, (d) CS-DML3, (e) CS-DMC5, and (f) CS-DML5, respectively. | S7 |
| **Figure S2** | EEM Profiles of raw sludge: (a) S-EPS, (b) LB-EPS, (c) TB-EPS fractions, respectively. | S8 |
| **Figure S3** | EEM Profiles of sludge S-EPS fraction treated by various flocculants under different dose (S-EPS samples were diluted by 10 times). | S9-S10 |
| **Figure S4** | EEM Profiles of sludge LB-EPS fraction treated by various flocculants under different dose (LB-EPS samples were diluted by 10 times). | S11-S12 |
| **Figure S5** | EEM Profiles of sludge TB-EPS fraction treated by various flocculants under different dose (TB-EPS samples were diluted by 10 times). | S13-S14 |
| **Figure S6** | The equipment diagram for sludge dewatering test. | S15 |
| **Reference** |  | S16-17 |

**Table S1** The residual TOC contents of various EPS fractions in sludge conditioned by different coagulants and flocculants.

| Samples | Dose  (mg/g TSS) | S-EPS  (mg/g TSS) | LB-EPS  (mg/g TSS) | TB-EPS  (mg/g TSS) |
| --- | --- | --- | --- | --- |
| Raw sludge |  | 2.841 | 22.65 | 34.29 |
| FeCl_3_ | 7.5 | 2.160 | 19.01 | 30.42 |
|  | 10.0 | 2.093 | 18.41 | 29.81 |
| CPAM | 7.5 | 1.804 | 16.22 | 26.21 |
|  | 10.0 | 1.831 | 16.19 | 26.84 |
| CS-DMC5 | 7.5 | 1.739 | 15.63 | 25.27 |
|  | 10.0 | 1.714 | 15.56 | 25.05 |
| CS-DML5 | 7.5 | 1.648 | 15.02 | 25.11 |
|  | 10.0 | 1.681 | 15.43 | 25.52 |

**Table S2** Characteristics of sludge.

| **Parameter*** | **Value** | **Parameter*** | **Value** |
| --- | --- | --- | --- |
| Moisture content (%) | 98.5 | Sr (mg/L) | 0.322 |
| pH | 6.9~7.2 | Cu (mg/L) | < 0.05 |
| VSS/TSS | 48.6~56.4% | Fe (mg/L) | < 0.05 |
| SRF (×10^12^ m/kg) | 9.07~11.25 | Cd (mg/L) | < 0.05 |
| Zeta potential (mV) | -(10.7~11.8) | Al (mg/L) | < 0.05 |
| SCOD (mg/L) | 54~73 | Mn (mg/L) | < 0.05 |
| Conductivity (mS/cm) | 0.50~0.71 | Ba (mg/L) | < 0.05 |
| Na (mg/L) | 44.9 | Ni (mg/L) | < 0.05 |
| Ca (mg/L) | 60.4 | Co (mg/L) | < 0.05 |
| Mg (mg/L) | 12.3 | Cr (mg/L) | < 0.05 |
| K (mg/L) | 10.3 | Zn (mg/L) | < 0.05 |
| Si (mg/L) | 0.726 |  |  |

*The moisture content and the ratio of volatile suspended solids (VSS) to total suspended solids (TSS, VSS/TSS) were analyzed according to APHA (1998)^3^; the pH of the filtrate was measured using a Delta 320 pH meter (Mettler Toledo, Switzerland); the zeta potential and conductivity were detected by a Zetasizer Nano Z (Malvern, UK); COD was detected by K_2_Cr_2_O_7_ titration method; Dissolved inorganic cations were using an optima 5300DV ICP-MS (PE, USA); SRF was measured according to experimental method section.

**Text S1** The measurement methods of bound water content.

The method for the measurement of bound water content in filter cakes after sludge dewatering was based on the following assumptions^6^: bound water could not freeze down to a threshold temperature, the heat required in the heating stage is equal to the latent heat absorbed for freezing free water, and the standard value of 334.7 J/g for the heat of phase change is accepted^7^. In this study, the water that could not freeze at -20 °C was determined as bound water. The sludge sample temperature was first decreased to -20 °C and then brought back to 20 °C at a rate of 2 °C/min. The sample mass was in the range of 2.0–6.0 mg.

**Text S2** The extraction method of EPS.

The extraction process of EPS is as follows^8,9^. Firstly, the sludge was centrifuged at 3000 rpm (764.7 *g*) for 10.0 min, and the supernatant was collected and regarded as S-EPS. Secondly, the sludge pellet was resuspended in 10 mL of 0.05% NaCl solution, sonicated at 20 kHz for 2.0 min, shaken horizontally at 150 rpm for 10.0 min, and sonicated again for another 2.0 min. Then the liquor was centrifuged at 5000 rpm (2124 *g*) for 10.0 min, and the collected supernatant was LB-EPS. [Finally](javascript:;), the pellet was resuspended in 0.05% NaCl solution again, sonicated for 3.0 min, heated at 60 °C for 30.0 min, and centrifuged at 8000 rpm (5438 *g*) for 10.0 min. The obtained supernatant was regarded as TB-EPS.

**Figure S1** The zeta potentials and corresponding FCMC and SRF of sludge conditioned by (a) CS-DMC1, (b) CS-DML1, (c) CS-DMC3, (d) CS-DML3, (e) CS-DMC5, and (f) CS-DML5, respectively.

**Figure S2** EEM Profiles of raw sludge: (a) S-EPS, (b) LB-EPS, (c) TB-EPS fractions, respectively.

|  | 1.5mg/g TSS | 3 mg/g TSS | 5 mg/g TSS | 7.5 mg/g TSS | 10 mg/g TSS | 14 mg/g TSS |
| --- | --- | --- | --- | --- | --- | --- |
| CS-DMC1 | 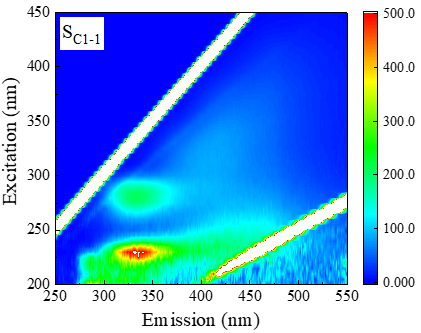 | 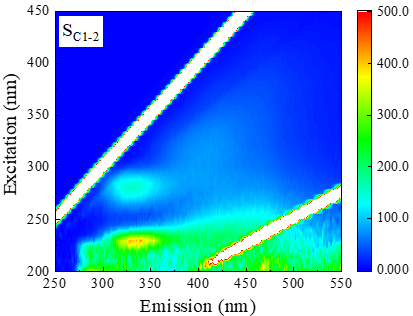 | 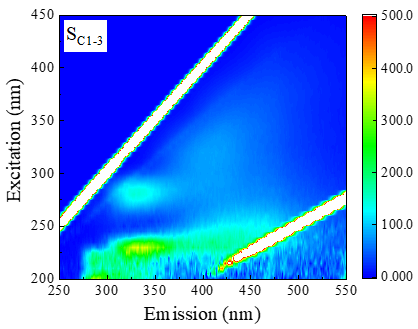 | 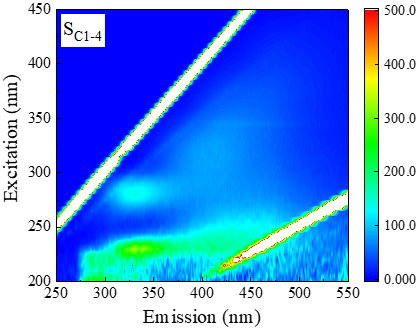 | 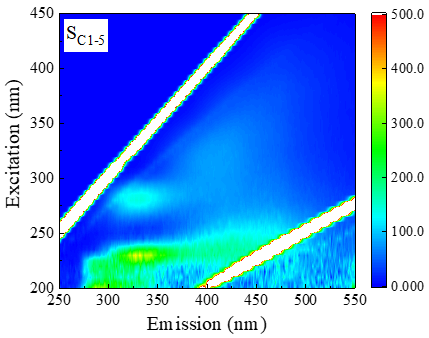 | 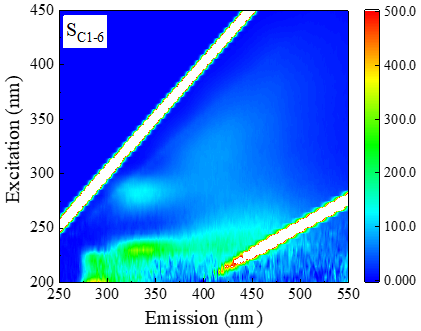 |
| CS-DML1 | 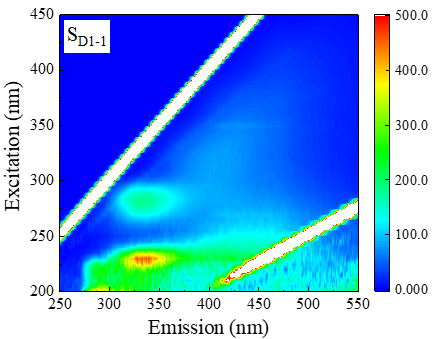 | 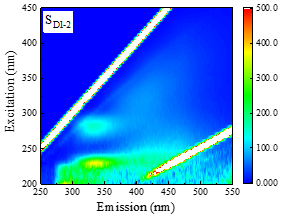 | 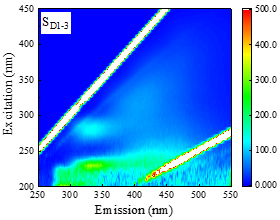 | 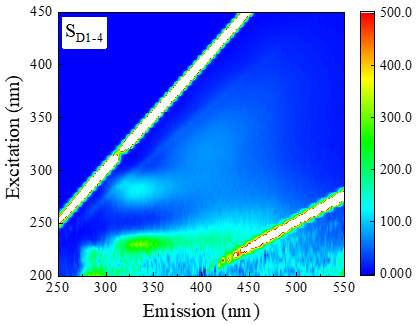 | 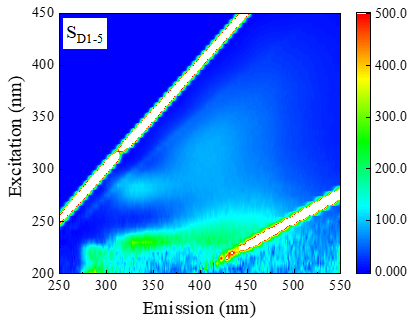 | 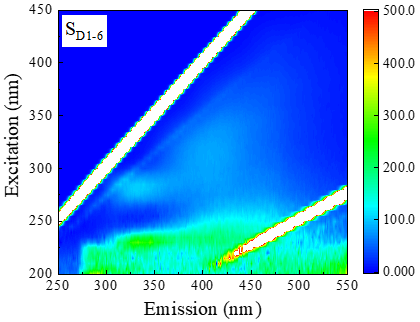 |
| CS-DMC3 | 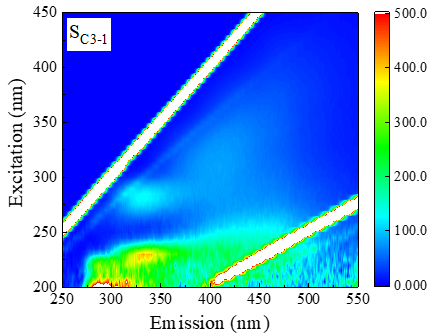 | 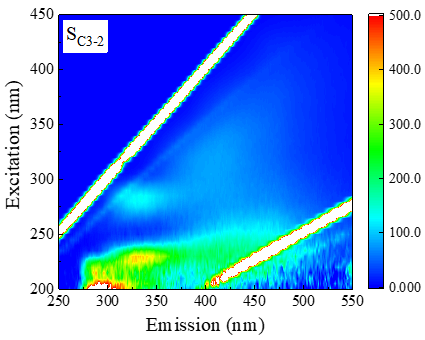 | 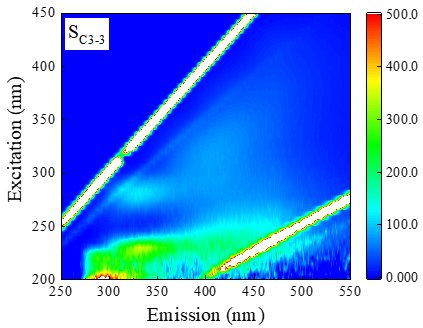 | 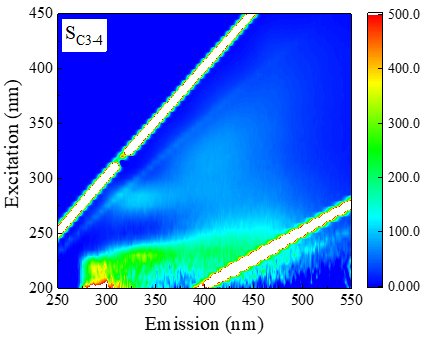 | 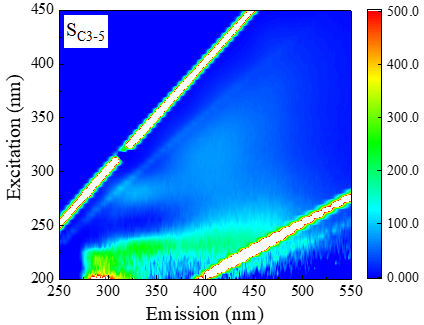 | 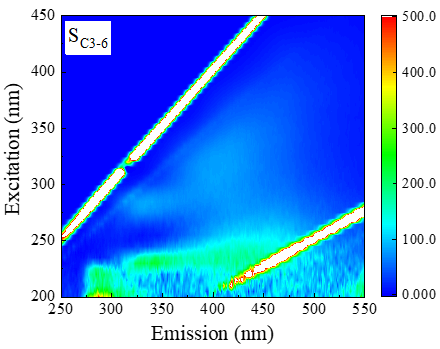 |
| CS-DML3 | 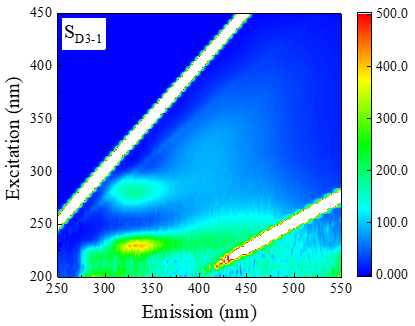 | 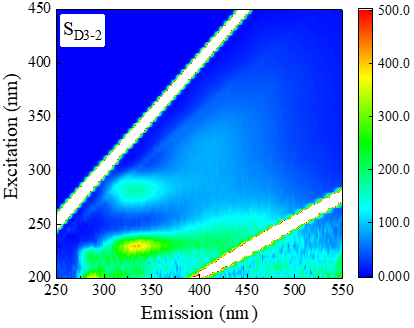 | 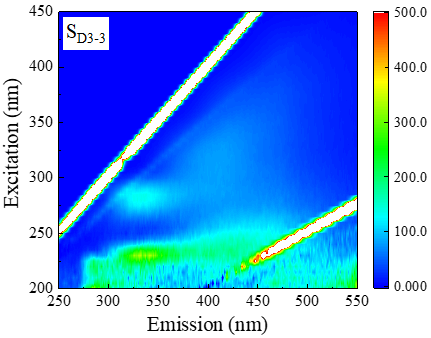 | 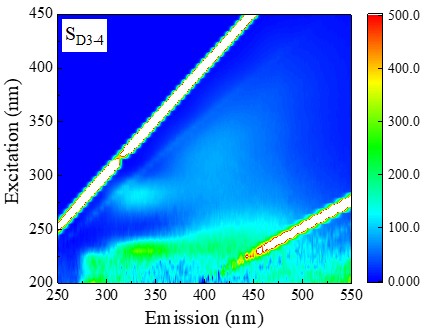 | 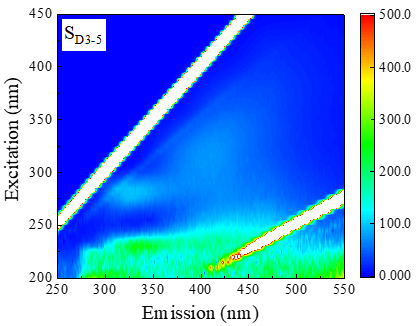 | 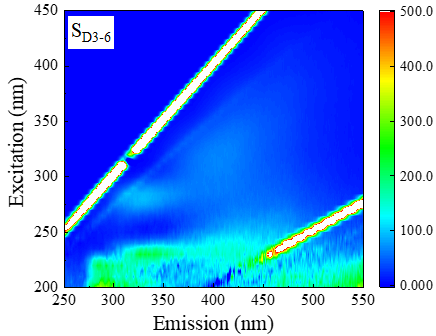 |
| CS-DMC5 | 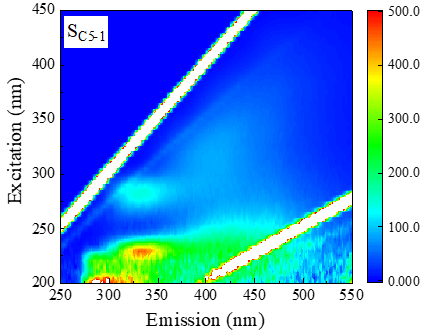 | 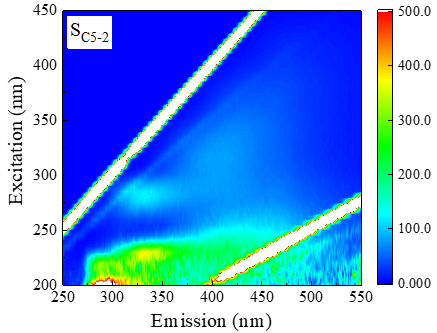 | 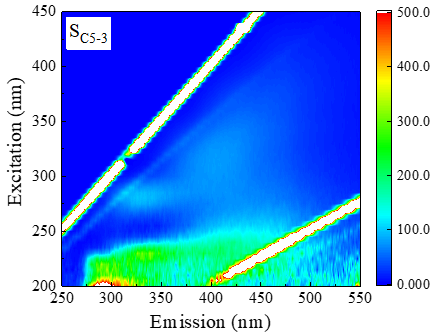 | 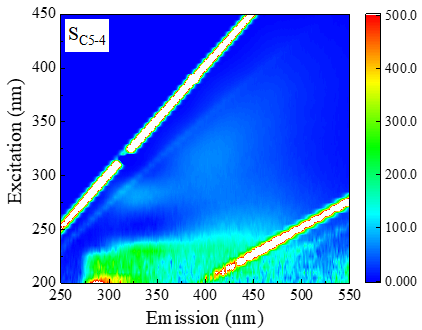 | 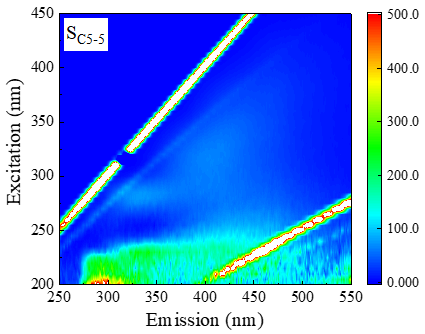 | 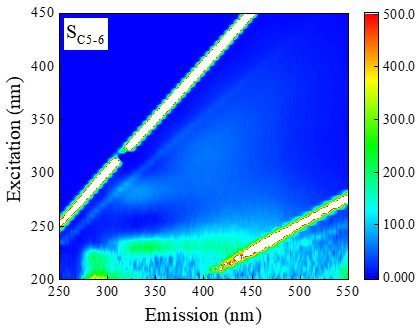 |
| CS-DML5 | 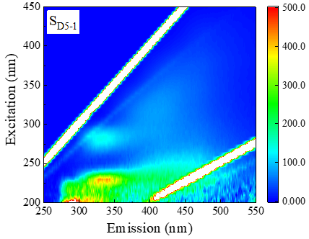 | 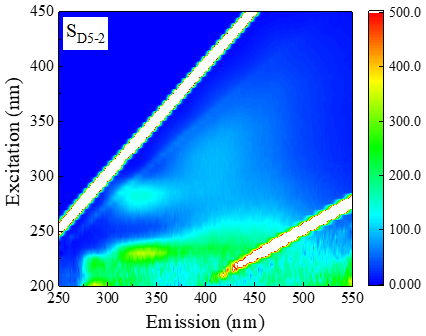 | 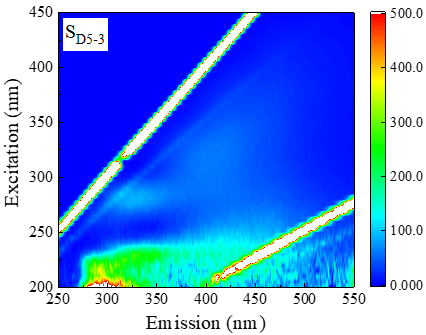 | 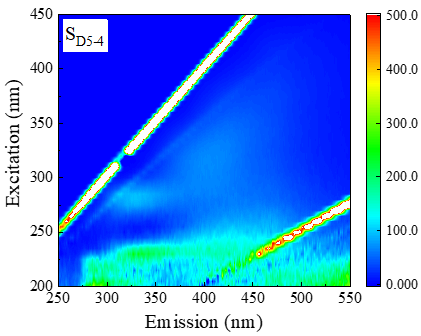 | 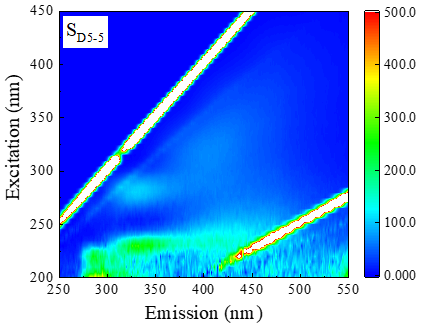 | 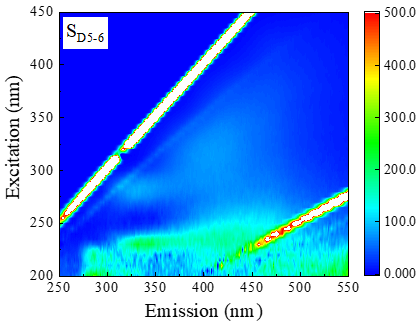 |

**Figure S3** EEM Profiles of sludge S-EPS fraction treated by various flocculants under different dose (S-EPS samples were diluted by 10 times).

|  | 1.5mg/g TSS | 3 mg/g TSS | 5 mg/g TSS | 7.5 mg/g TSS | 10 mg/g TSS | 14 mg/g TSS |
| --- | --- | --- | --- | --- | --- | --- |
| CS-DMC1 | 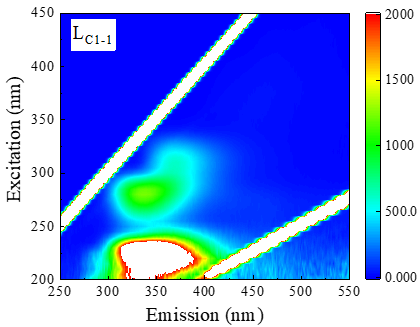 | 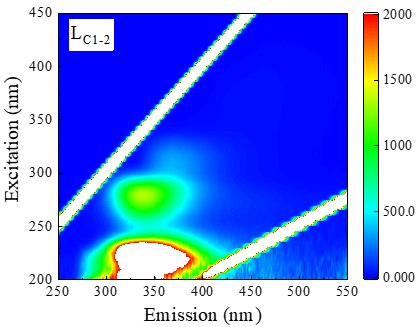 | 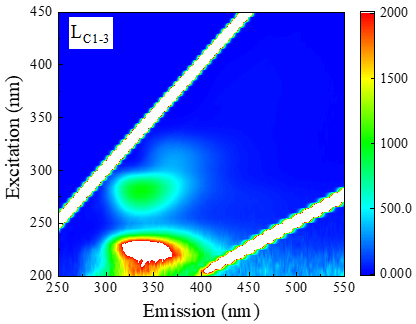 | 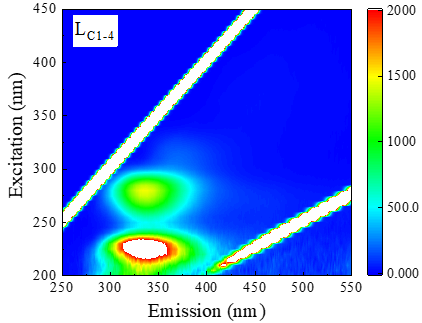 | 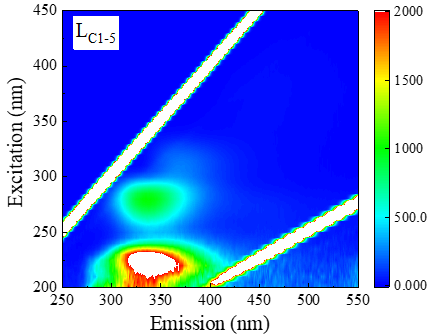 | 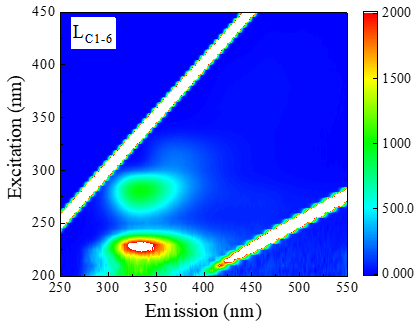 |
| CS-DML1 | 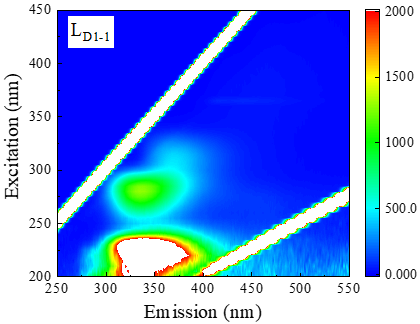 | 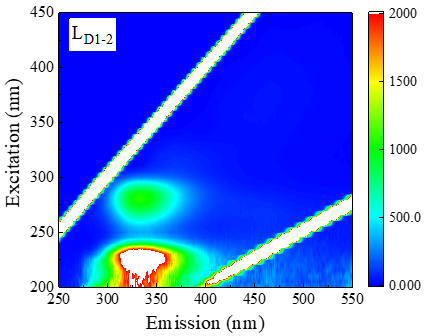 | 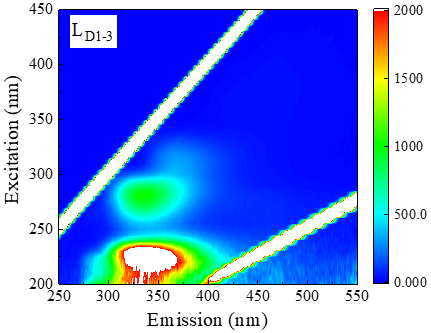 | 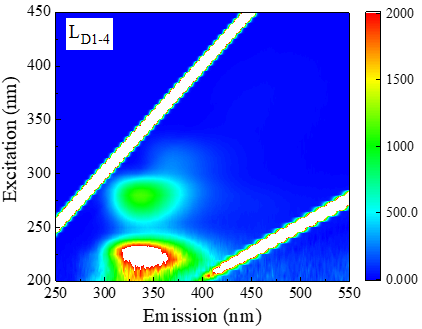 | 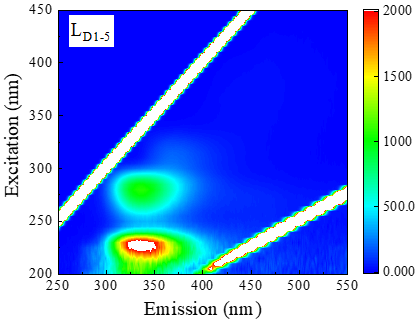 | 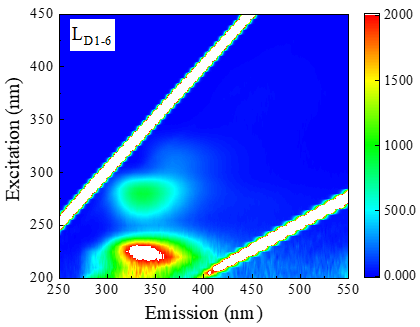 |
| CS-DMC3 | 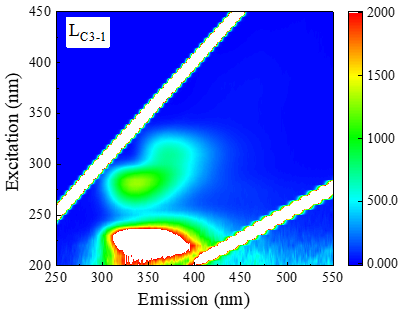 | 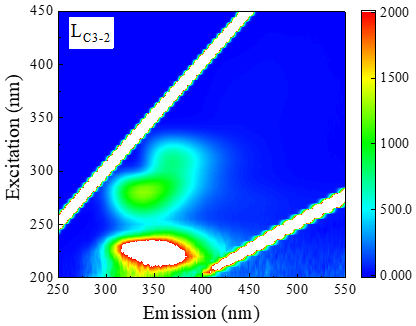 | 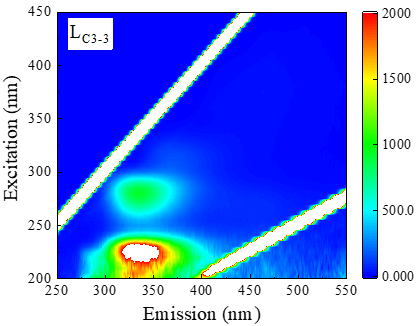 | 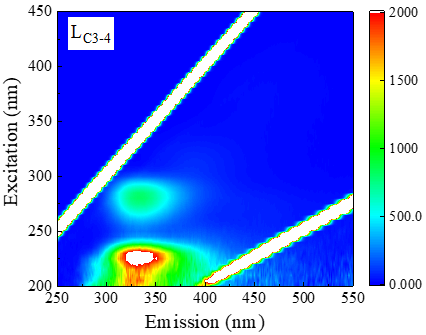 | 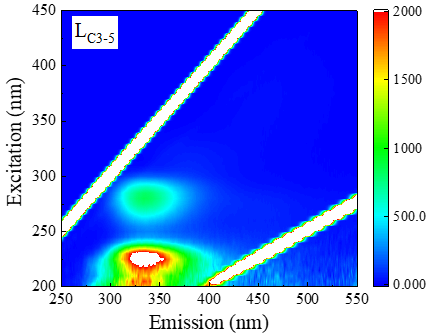 | 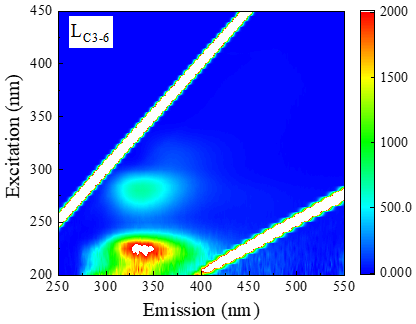 |
| CS-DML3 | 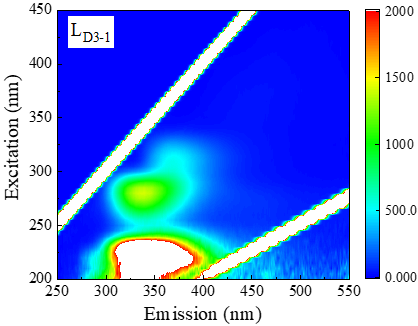 | 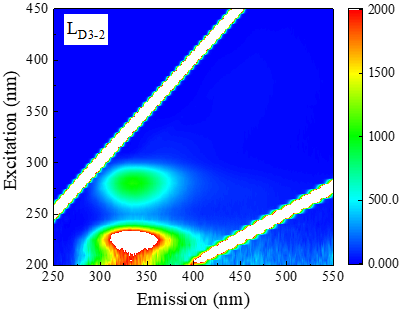 | 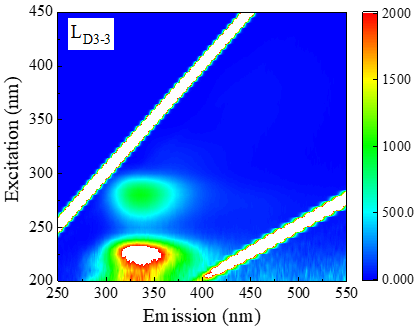 | 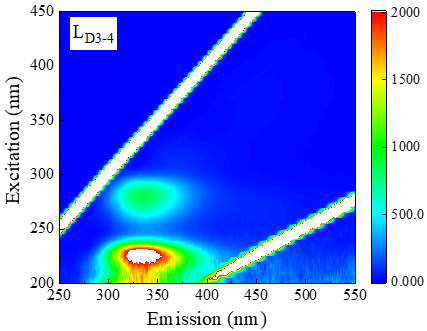 | 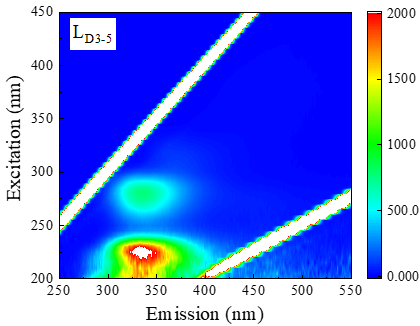 | 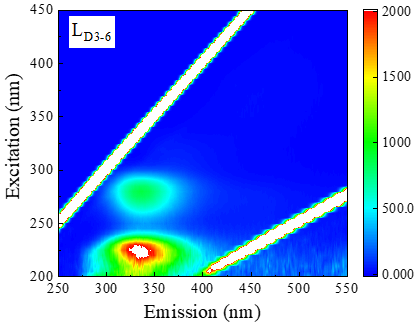 |
| CS-DMC5 | 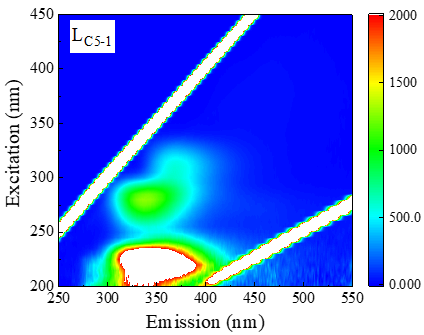 | 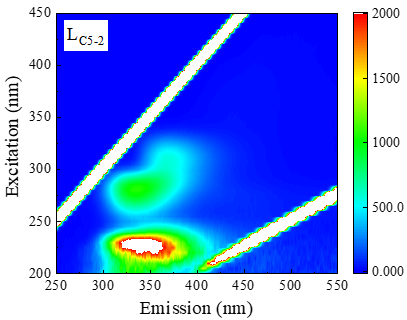 | 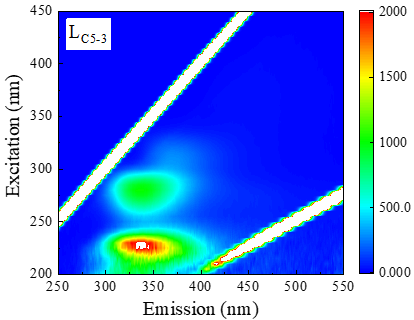 | 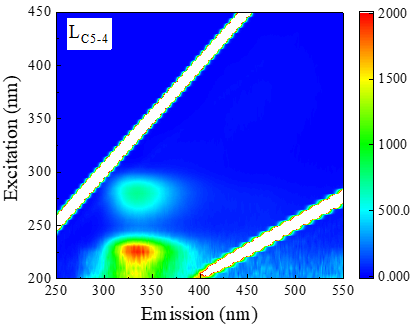 | 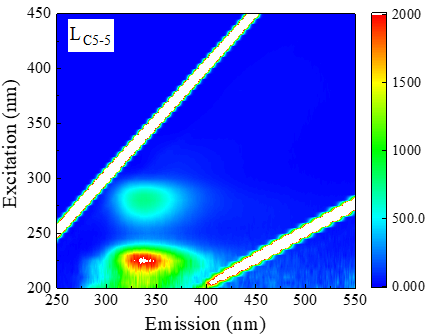 | 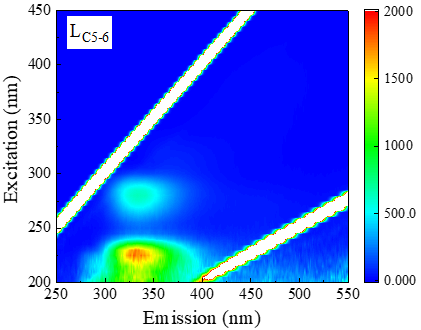 |
| CS-DML5 | 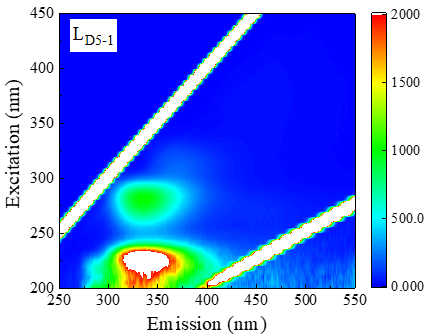 | 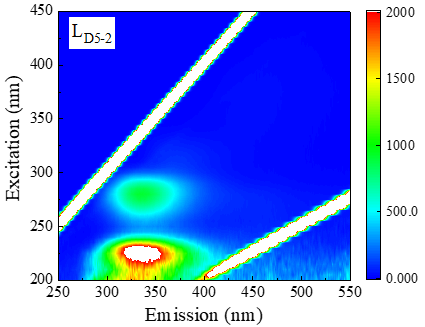 | 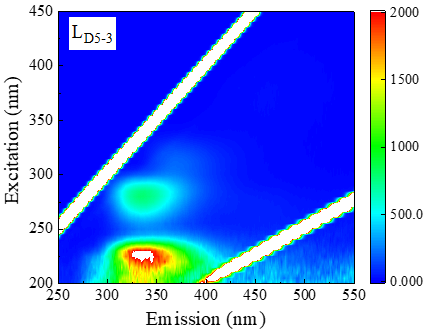 | 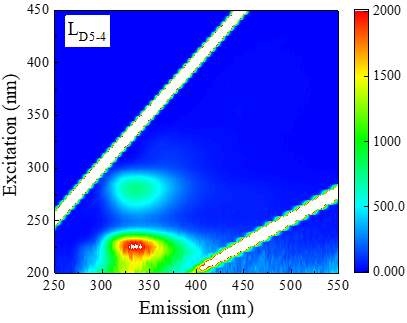 | 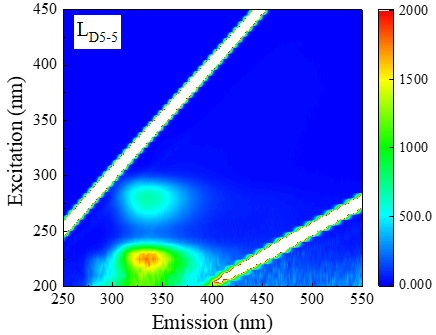 | 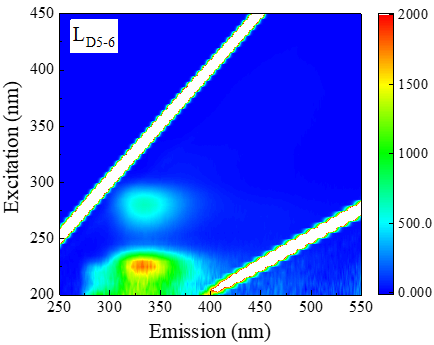 |

**Figure S4** EEM Profiles of sludge LB-EPS fraction treated by various flocculants under different dose (LB-EPS samples were diluted by 50 time).

|  | 1.5 mg/g TSS | 3 mg/g TSS | 5 mg/g TSS | 7.5 mg/g TSS | 10 mg/g TSS | 14 mg/g TSS |
| --- | --- | --- | --- | --- | --- | --- |
| CS-DMC1 | 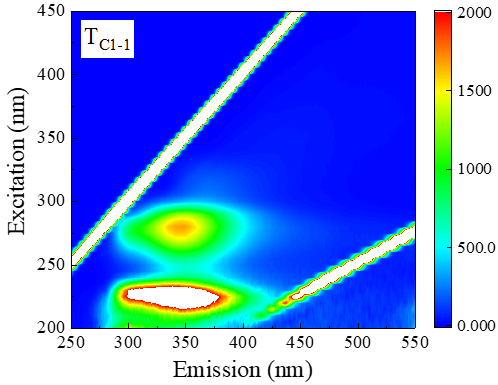 | 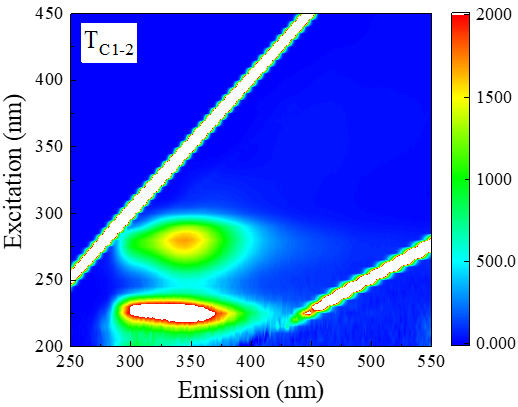 | 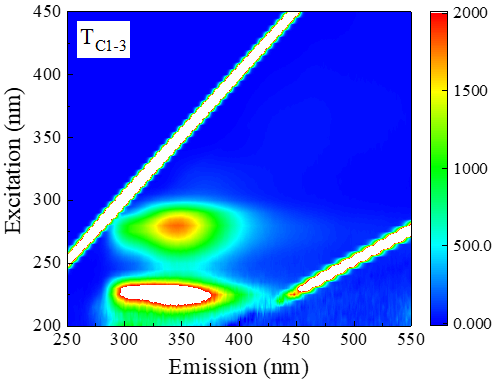 | 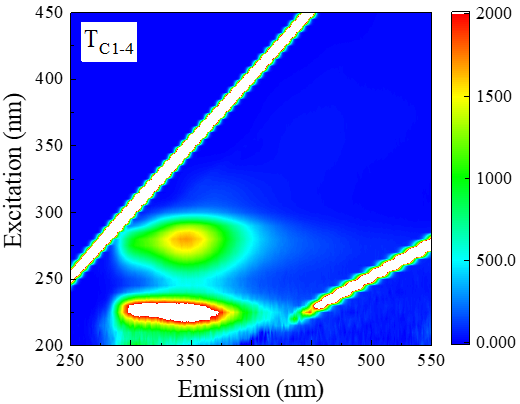 | 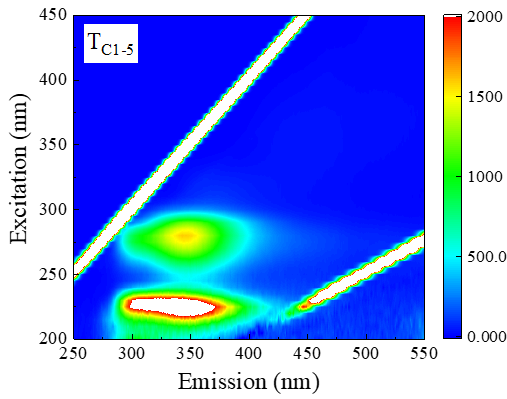 | 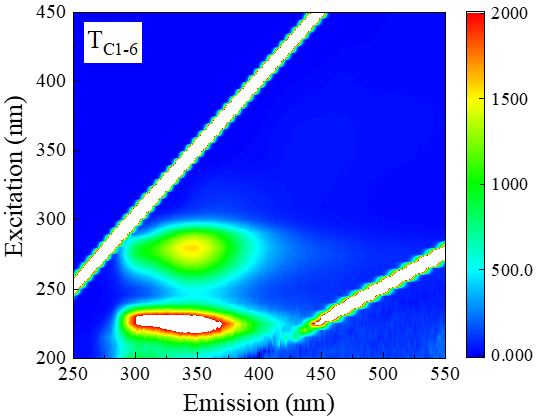 |
| CS-DML1 | 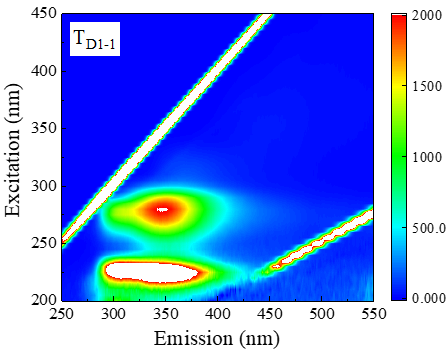 | 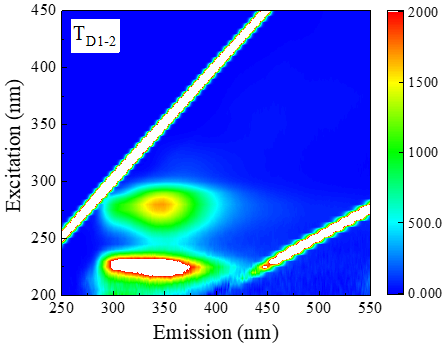 | 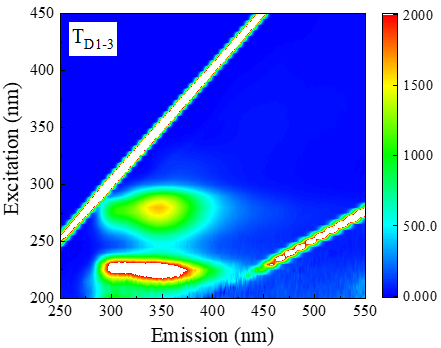 | 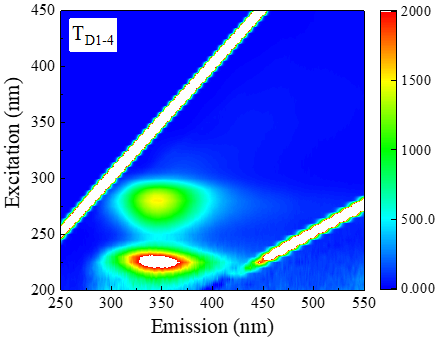 | 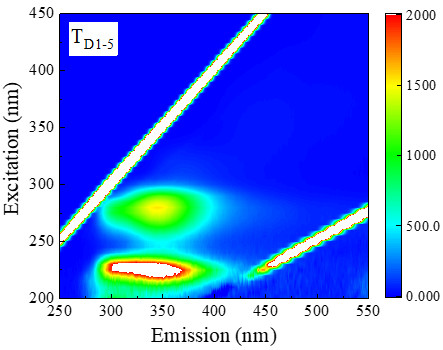 | 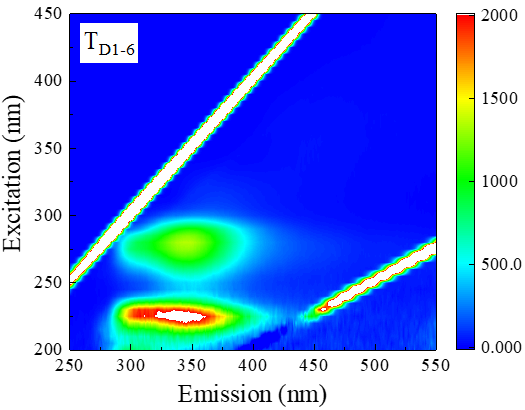 |
| CS-DMC3 | 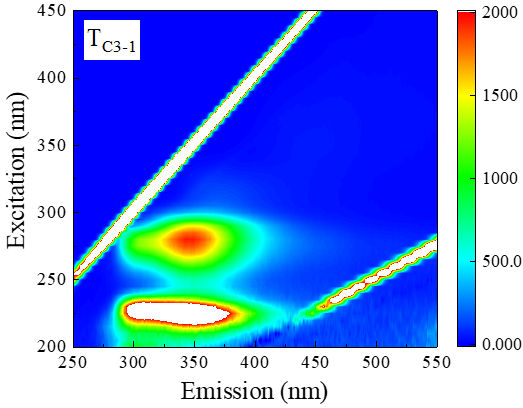 | 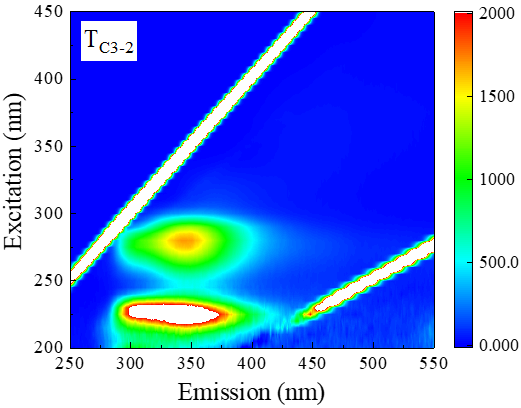 | 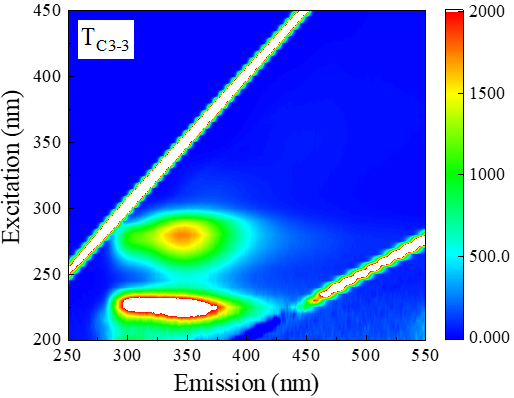 | 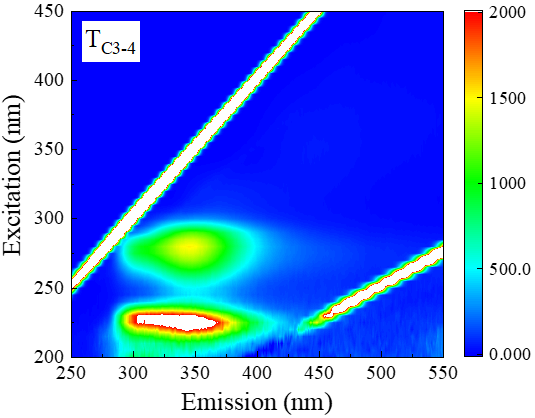 | 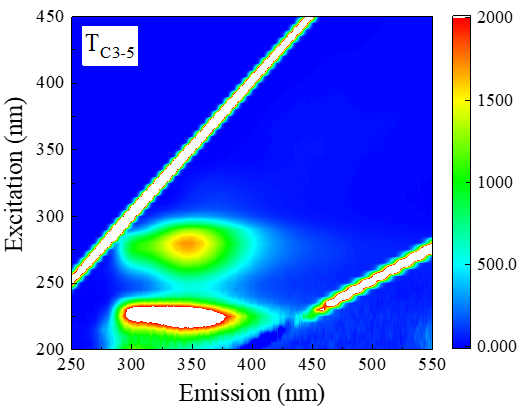 | 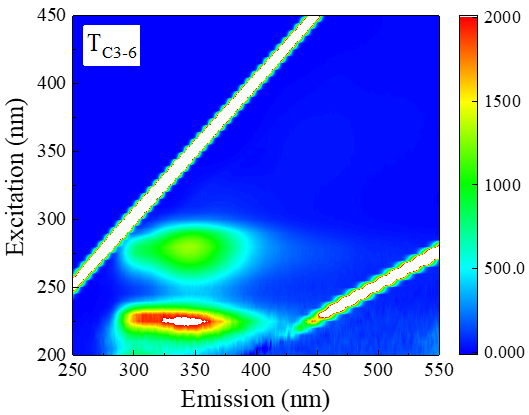 |
| CS-DML3 | 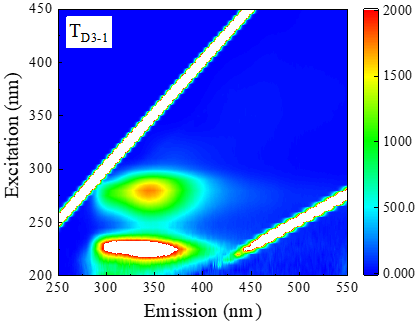 | 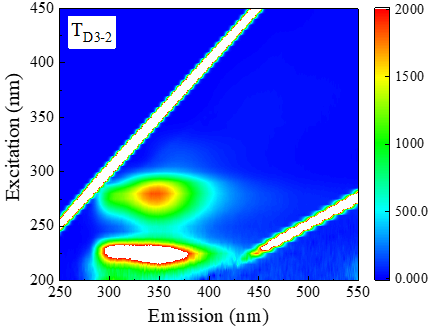 | 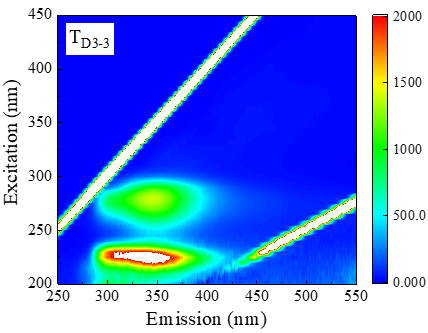 | 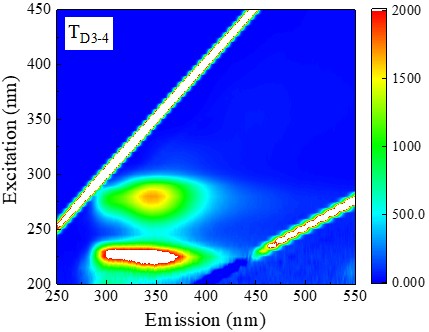 | 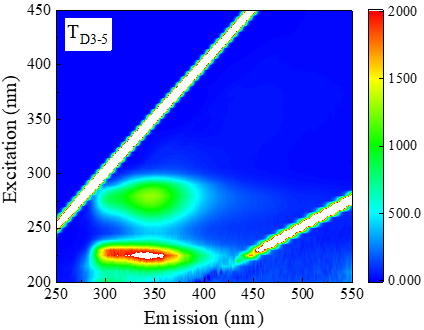 | 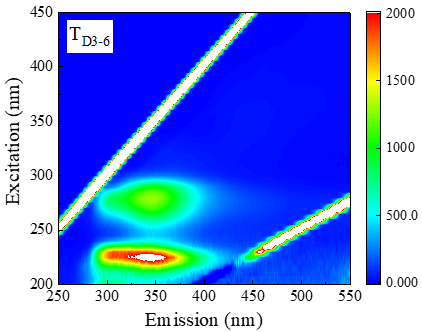 |
| CS-DMC5 | 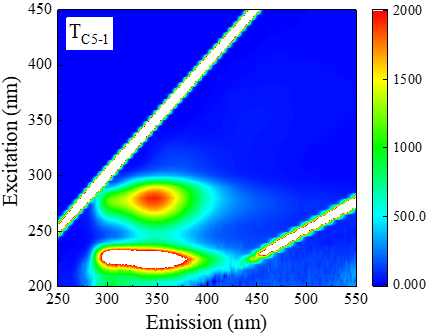 | 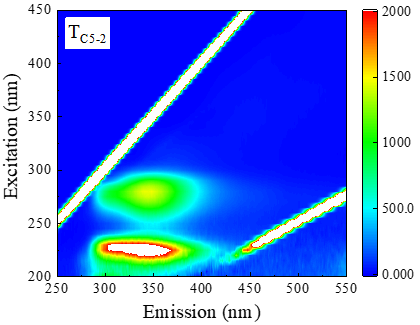 | 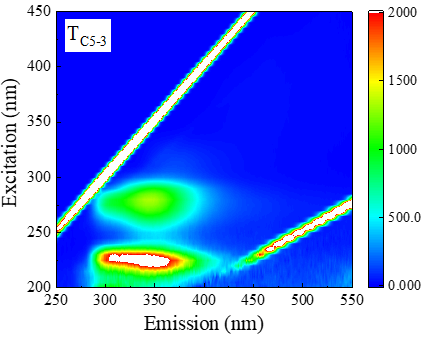 | 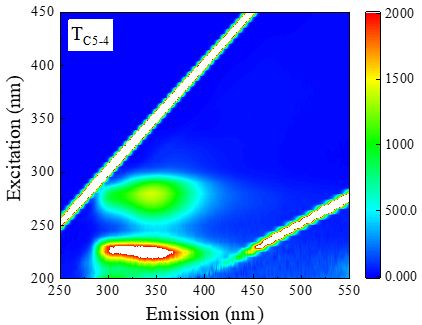 |  |  |
| CS-DML5 |  |  |  |  |  |  |

**Figure S5** EEM Profiles of sludge TB-EPS fraction treated by various flocculants under different dose (TB-EPS samples were diluted by 100 times).

**Figure S6** The equipment diagram for sludge dewatering test.

**Reference**

1. Chen, Z., Zhang, W.J., Wang, D.S., Ma, T. Bai, R.Y. Enhancement of activated sludge dewatering performance by combined composite enzymatic lysis and chemical reflocculation with inorganic coagulants: kinetics of enzymatic reaction and re-flocculation morphology, Water Res. **83**, 367–376 (2015).

2. Wei, H., Ren, J., Li, A.M., Yang, H. Sludge dewaterability of a starch-based flocculant and its combined usage with ferric chloride. Chem. Eng. J. **349**, 737-747

(2018).

3. APHA. Standard Methods for the Examination of Water and Wastewater, twentiethed. American Public Health Association, American Water Work Association, Water Environment federation, Washington (1998).

4. Cheng, R. S., Extrapolation of viscosity data and calculation of intrinsic viscosity from one concentration of solution viscosity, Polym. Bull. Chin. **3**, 159-163 (1960).

5. Williams, P. A., Handbook of Industrial Water Soluble Polymers. Blackwell Publishing Ltd., UK, (2007

6. Morgan, J. W., Forster, C. F. & Evison, L. A comparative study of the nature of biopolymers extracted from anaerobic and activated sludges. Water Res. **24**(6), 743-750 (1990).

7. Niu, M., Zhang, W., Wang, D., Chen, Y. & Chen, R. Correlation of physicochemical properties and sludge dewaterability under chemical conditioning using inorganic coagulants. Bioresource Technol. **144**, 337-343 (2013).

8. [Li, Y. F](http://apps.webofknowledge.com/OutboundService.do?SID=6EuSy63SivTFB8jL7ic&mode=rrcAuthorRecordService&action=go&product=WOS&lang=zh_CN&daisIds=41168362)., [Xu, Q. X](http://apps.webofknowledge.com/OutboundService.do?SID=6EuSy63SivTFB8jL7ic&mode=rrcAuthorRecordService&action=go&product=WOS&lang=zh_CN&daisIds=1391951)., [Liu, X. R](http://apps.webofknowledge.com/OutboundService.do?SID=6EuSy63SivTFB8jL7ic&mode=rrcAuthorRecordService&action=go&product=WOS&lang=zh_CN&daisIds=8303642)., [Wang, Y. L](http://apps.webofknowledge.com/OutboundService.do?SID=6EuSy63SivTFB8jL7ic&mode=rrcAuthorRecordService&action=go&product=WOS&lang=zh_CN&daisIds=3912962).,[Wang, D. B](http://apps.webofknowledge.com/OutboundService.do?SID=6EuSy63SivTFB8jL7ic&mode=rrcAuthorRecordService&action=go&product=WOS&lang=zh_CN&daisIds=115184)., [Yang, G. J](http://apps.webofknowledge.com/OutboundService.do?SID=6EuSy63SivTFB8jL7ic&mode=rrcAuthorRecordService&action=go&product=WOS&lang=zh_CN&daisIds=254096)., [Yuan, X. Z](http://apps.webofknowledge.com/OutboundService.do?SID=6EuSy63SivTFB8jL7ic&mode=rrcAuthorRecordService&action=go&product=WOS&lang=zh_CN&daisIds=29048804)., [Yang, F](http://apps.webofknowledge.com/OutboundService.do?SID=6EuSy63SivTFB8jL7ic&mode=rrcAuthorRecordService&action=go&product=WOS&lang=zh_CN&daisIds=3889648)., [Huang, J](http://apps.webofknowledge.com/OutboundService.do?SID=6EuSy63SivTFB8jL7ic&mode=rrcAuthorRecordService&action=go&product=WOS&lang=zh_CN&daisIds=41596961). & [Wu, Z. B](http://apps.webofknowledge.com/OutboundService.do?SID=6EuSy63SivTFB8jL7ic&mode=rrcAuthorRecordService&action=go&product=WOS&lang=zh_CN&daisIds=250615). Peroxide/Zero-valent iron (Fe^0^) pretreatment for promoting dewaterability of anaerobically digested sludge: A mechanistic study. J. Hazard. Mater. **400**, 123112 (2020).

9. Lee, D. J. & Lee, S. F. Measurement of bound water content in sludge: the use of differential scanning calorimetry (DSC). J. Chem. Technol. Biotechnol. **62** (4), 359–365 (1995).
